# Aspulvinones Suppress Postprandial Hyperglycemia as Potent α-Glucosidase Inhibitors From *Aspergillus terreus* ASM-1

**DOI:** 10.3389/fchem.2021.736070

**Published:** 2021-08-17

**Authors:** Changjing Wu, Xiang Cui, Luzhen Sun, Jiajia Lu, Feng Li, Minghui Song, Yunxia Zhang, Xinqi Hao, Congkui Tian, Maoping Song, Xiaomeng Liu

**Affiliations:** ^1^ College of Life Sciences and Agronomy, Zhoukou Normal University, Zhoukou, China; ^2^ College of Chemistry and Molecular Engineering, Zhengzhou University, Zhengzhou, China; ^3^ Wuling Mountain Institute of Natural Medicine, Hubei Minzu University, Enshi, China; ^4^ College of Public Health, Xinxiang Medical University, Xinxiang, China

**Keywords:** *Aspergillus terreus*, secondary metabolites, aspulvinone, structure elucidation, α-glucosidase inhibitory effect

## Abstract

Chemical investigation of *Aspergillus terreus* ASM-1 fermentation resulted in the isolation of three new prenylated aspulvinones V–X (**1**–**3**), together with the previously reported analogs, aspulvinone H (**4**), J-CR (**5**), and R (**6**). Their structures were elucidated by various spectroscopic methods including HRESIMS and NMR, and the absolute configurations of **2** and **3** were determined by ECD comparison. Compounds **1**–**6** were evaluated for α-glucosidase inhibitory effects with acarbose as positive control. As a result, compounds **1** and **4** exhibited potent α-glucosidase inhibitory activities with IC_50_ values of 2.2 and 4.6 µM in mixed-type manners. The thermodynamic constants recognized the interaction between inhibitors and α-glucosidase was hydrophobic force-driven spontaneous exothermic reaction. The CD spectra also indicate that the compounds **1** and **4** changed the enzyme conformation. Furthermore, compound **4** significantly suppressed the increases in postprandial blood glucose levels in the C57BL/6J mice.

## Introduction

Diabetes mellitus is chronic metabolic disease with worldwide concerns, which causes a major challenge for the health system (Kharroubi and Darwish, 2015). The high prevalence of diabetes has focalized much efforts for novel therapeutic alternatives (Ghosh et al., 2016). Nowadays, alleviating postprandial hyperglycemia is one of the first-line therapeutical strategies for the treatment of diabetes and its complications (Taylor et al., 2021). α-Glucosidase inhibitors (AGIs), such as acarbose, miglitol, and voglibose, are usually employed for controlling postprandial blood glucose levels by delaying the intestinal digestion of carbohydrates (Hossain et al., 2020). However, utilization of clinical AGIs often have some shortcomings such as side-effects including abdominal discomfort and flatulence, limited efficacy, failure in metabolism adjustment (Calcutt et al., 2009). Therefore, much effort has been focused on searching for natural AGIs with better safety and efficacy from natural sources in the past decade (Deng et al., 2015; Zhang et al., 2020).

**FIGURE 1:**
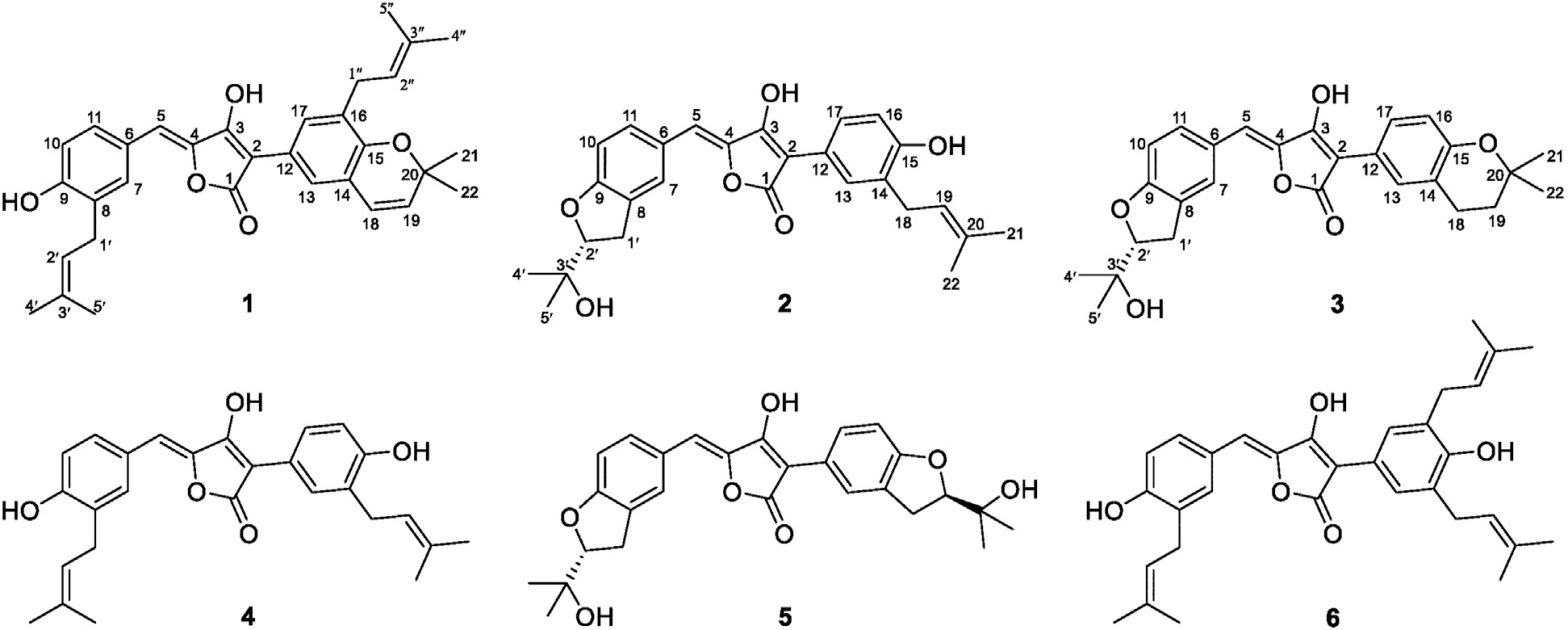
Chemical structures of Compounds **1**–**6**.

*Aspergillus terreus* ML-44 is marine-derived fungi previously isolated from the fresh gut of pacific oyster. Our former study reported five terretonins isolated from ML-44 fermentation, including a new one, which showed weak anti-inflammatory activity (Wu et al., 2019). In order to exploit the potential of strain ML-44 in the medical field, the diethyl sulfate (DES) mutagenesis strategy (Fang et al., 2014) was applied to strain ML-44 in this study, mutant strain ASM-1 was screened out with different phenotypic morphology of colonies. HPLC-DAD-UV analysis of the mutant fermentations comparing to parent strain exhibited a series of metabolites with unique ultra-violet absorption were observed in the mutant ASM-1. Subsequent HPLC-guided chemical investigation of ASM-1 fermentation resulted in the isolation of six aspulvinone derivatives (**1**–**6**), including three new ones (**1**–**3**) (Figure 1). Aspulvinones and the analogs have been reported with various biological activities, such as inhibiting antibacterial (Machado et al., 2021), luciferase (Cruz et al., 2011), anti-influenza A viral (Gao et al., 2013), anticancer (Sun et al., 2019), anti-DPPH radicals (Zhang et al., 2015), as well as α-glucosidase inhibitory activity (Dewi et al., 2014; Wang et al., 2016; Zhang et al., 2016). However, there has been no systematic report on the mechanism for inhibition of α-glucosidase, structure–activity relationships, and hpyerglycemic effect *in vivo* by natural aspulvinones. In this study, the α-glucosidase inhibitory activities of compounds **1**–**6** were evaluated *in vitro*, in silico, and *in vivo*. Herein, we report the isolation, structure elucidation, and the α-glucosidase inhibitory activities of the isolated aspulvinones.

## Material and Methods

### General Experimental Procedures

Sephadex™ LH-20 (GE Healthcare, Uppsala, Sweden), and YMC*GEL^®^ ODS-A-HG (12 nm S-50 μm, YMC Co., Ltd., Kyoto, Japan) were used for column chromatography. The MPLC was performed on a QuikSep chromatographic system (H&E, Beijing, China), and a Gemini C18 column (21.2 × 250 mm, column temperature: 26°C) was used for separation and purification. Optical rotations were measured on a JASCO P-2000 digital polarimeter (JASCO, Tokyo, Japan). UV spectra were recorded on a PerkinElmer Lambda 25 spectrophotometer. Electronic Circular Dichroism (ECD) data were taken on a Chirascan circular dichroism spectrometer (Applied Photophysics, Surrey, United Kingdom). HR-ESI-MS was measured on Agilent 6520 Q-TOP mass spectrometer (Agilent, CA, United States), and all 1D and 2D NMR spectra were obtained on a Bruker-500 (500 MHz ^1^H and 125 MHz ^13^C-NMR) NMR spectrometer. A SynergyHTX micro plate reader (BioTek, VT, United States) was used to read optical density (OD). The intrinsic fluorescence spectra (280–500 nm) were measured using Perkin Elmer LS55 fluorescence spectrophotometer (United Kingdom).

### Chemical Mutagenesis of *A. terreus* ML-44 and Mutant Selection

The DES mutagenesis procedure was referred to the method that we previously reported (Fang et al., 2014), and with proper modifications: DES was dissolved in DMSO to obtain a 20% (v/v) solution, which was further mixed with spore suspension of *A. terreus* ML-44 in a ratio of 1:9 (v/v). The mixture was treated with assistance of ultrasonic wave (40 KHz) at room temperature. Each 80 μl portion of the treated spore suspensions was sampled and spread on PDA plates at 1 and 2 h of treatment followed by incubation at 28°C for 5–7 days. Mutants from the test groups were obtained by selection of colonies with different colonial morphology, and the genetic stability were verified by passing three generations.

The initial ML-44 strain and mutants were activated by incubation at 28°C for 3–5 days, and further inoculated into 100 ml of liquid medium (glucose 2%, maltose 1%, mannitol 2%, glutamic acid 1%, peptone 0.5%, and yeast extract 0.3% in distilled water) in an Erlenmeyer (250 ml) and fermented at 28°C on a rotary shaker at 200 rpm for 12 days. Each 100 ml of the fermentation broth was extracted with equal volumes of EtOAc with assistance of ultrasonic wave (40 KHz) for 30 min. The EtOAc extractions were concentrated in vacuo at 37°C, followed by re-dissolved in 1.0 ml methanol which were used for further chemical analysis. HPLC-PDAD-UV analysis was performed using an analytical Kromasil C18 column (5 μm, 100 Å, 4.6 × 250 mm; Akzo Nobel) on an Agilent 1100 HPLC system equipped with photo-diode array detector (G1316A). After filtered with 0.45 μm membrane, the extraction solution in methanol (1.0 ml) was injected (10 μl) into the column and eluted with a MeOH-H_2_O linear gradient (20%→100% MeOH in 30 min followed by 5 min with isocratic 100% MeOH) mobile phase (flow rate 1 ml/min). The acquired PDAD data were processed with Agilent OpenLAB software.

### Chemical Investigation to Mutant ASM-1

The mutant ASM-1 was inoculated into ten Erlenmeyer (500 ml) each containing 200 ml of sterile liquid medium and cultured at 28°C for 48 h on a rotary shaker at 200 rpm providing a seed culture (2 L). The seed culture was inoculated into a fermentation cylinder containing the same sterile liquid medium (70 L), and was cultured at 28°C for 12 days, with sterile compressed air passes from the bottom of the cylinder keeping a positive pressure of 0.15 MPa. The whole broth (65 L) was filtered to separate into the filtrate and the mycelial cake. The filtrate (60 L) was subjected to an AB-8 macroporous resin column (column volume, CV 2.4 L), eluted by water and 95% ethanol successively. The water elute (3 CVs) was discarded, and the 95% ethanol elute (3 CVs) was gathered. The mycelial cake was extracted two times with 95% ethanol (5 L) assisted by ultra-sonication for 2 h, followed by filtration giving the ethanol extract. All the ethanol solutions were combined and concentrated to a water suspension, which was further extracted with EtOAc to afford a total of 60.5 g of EtOAc extract.

The EtOAc extract (60.5 g) of the mutant ASM-1 was subjected to silica gel column chromatography by stepwise elution with b. p. 60–90°C petroleum ether (P)- dichloro-methane (D)-methanol (M) to obtain 9 fractions. HPLC analysis showed that the newly produced metabolites were contained in Fr-4 (1.9 g, eluted by D), Fr-5 (5 g, eluted by DM 98:2), and Fr-7 (2.3 g, eluted by DM 90:10) (Supplementary Figure S3). Subsequent repeated preparative reverse phase HPLC separation led to the purification of compounds. Fr-4 was subjected to reduced pressure ODS column chromatography (cc) to give subfraction Fr-4–8 (0.3 g, eluted by 90%M), which was further separated by HPLC (Methanol-H_2_O (0.1%HCl) 90:10, 10 ml/min) to afford **1** (25 mg, *t*_R_ = 36.0 min) and **6** (2.1 mg, *t*_R_ = 24.5 min); Fr-5–4 (1.6 g, eluted by 80%M), the subfraction of Fr-5 by ODS cc, afforded **3** (12 mg, *t*_R_ = 64.0 min) and **4** (218 mg, *t*_R_ = 67.5 min) with preparative HPLC separation (Methanol-H_2_O (0.1%HCl) 65:35, 10 ml/min); Fr-7 was separated by ODS cc to give subfraction Fr-7–9 (0.6 g, eluted by 80%M), which provided **2** (28 mg, *t*_R_ = 28.4 min) and **5** (16 mg, *t*_R_ = 18.6 min) after preparative HPLC separation (Methanol-H_2_O (0.1%HCl) 77:23, 10 ml/min).

Aspulvinone V (**1**): yellow solid (MeOH), UV (MeOH) λmax (log ε): 203 (4.36), 239 (4.11), 380 (4.23). Positive HR-ESI-MS: *m/z* measured 499.2480 [M + H]^+^, calcd for C_32_H_35_O_5_ [M + H]^+^ 499.2484. ^1^H NMR and ^13^C NMR spectroscopic data, see Table 1.

**TABLE 1:** 500 MHz ^1^H and 125 MHz^13^C NMR data of compounds **1**–**3**, **5** in CD_3_OD.^a^

No	1		2		3		5	
	*δ*_C_	*δ*_H_ (J in Hz)	*δ*_C_	*δ*_H_ (J in Hz)	*δ*_C_	*δ*_H_ (J in Hz)	*δ*_C_	*δ*_H_ (J in Hz)
1	171.2 s	—	171.3 s	—	171.2 s	—	171.1 s	—
2	102.2 s	—	102.7 s	—	102.4 s	—	102.6 s	—
3	163.5 s	—	163.1 s	—	163.3 s	—	163.3 s	—
4	141.5 s	—	141.8 s	—	141.8 s	—	141.8 s	—
5	109.5 d	6.37, s	108.9 d	6.36, s	109.1 d	6.38, s	109.1 d	6.39, s
6	125.8 s	—	127.1 s	—	127.0 s	—	127.1 s	—
7	133.4 d	7.50, brs	128.0 d	7.67, brs	128.1 d	7.68, brs	128.0 d	7.70, brs
8	129.8 s	—	129.7 s	—	129.7 s	—	129.8 s	—
9	157.6 s	—	162.2 s	—	162.2 s	—	162.2 s	—
10	116.1 d	6.79, d (8.6)	110.3 d	6.75, d (8.3)	110.3 d	6.76, d (8.4)	110.3 d	6.77, d (8.4)
11	130.8 d	7.49, brd (8.6)	132.5 d	7.46, brd (8.3)	132.5 d	7.46, brd (8.4)	132.5 d	7.47, brd (8.4)
12	123.2 s	—	122.2 s	—	122.0 s	—	123.2 s	—
13	124.8 d	7.41, d (2.1)	124.8 d	7.65, d (2.3)	130.1 d	7.61, d (2.1)	125.5 d	7.72, brs
14	122.2 s	—	129.1 s	—	122.5 s	—	128.6 s	—
15	151.1 s	—	155.7 s	—	154.7 s	—	160.7 s	—
16	130.0 s	—	115.5 d	6.78, d (8.3)	117.9 d	6.72, d (8.4)	109.7 d	6.77, d (8.4)
17	130.2 d	7.55, d (2.1)	127.6 d	7.56, dd (8.3, 2.3)	128.0 d	7.59, dd (8.4, 2.1)	129.0 d	7.64, brd (8.4)
18	123.7 d	6.36, d (9.7)	29.3 t	3.31, overlapped	23.4 t	2.81, t (6.8)	31.5 t	3.16–3.28, m
19	131.8 d	5.68, d (9.7)	123.9 d	5.35, brt (7.3)	33.8 t	1.82, t (6.8)	90.7 s	4.65, t-like (8.8)
20	77.4 s	—	133.0 s	—	75.5 s	—	72.5 s	—
21	28.2 q	1.40, s	26.0 q	1.75, brs	27.13 q	1.32, s	25.4 q	1.22, s
22	28.2 q	1.40, s	17.9 q	1.74, brs	27.11 q	1.32, s	25.18 q	1.26, s
1′	29.2 t	3.30, d (7.4)	31.2 t	3.20, dd (16.0, 8.5)	31.2 t	3.21, dd (16.0, 8.4)	31.2 t	3.16–3.28, m
				3.15, dd (16.0, 9.6)		3.16, dd (16.0, 9.4)		
2′	123.6 d	5.33, brt (7.4)	91.1 d	4.59, dd (9.6, 8.5)	91.1 d	4.61, dd (9.4, 8.4)	91.2 d	4.63, t-like (8.8)
3′	133.5 s	—	72.4 s	—	72.4 s	—	72.4 s	—
4′	26.0 q	1.76, brs	25.4 q	1.21, s	25.4 q	1.22, s	25.4 q	1.22, s
5′	17.9 q	1.74, brs	25.2 q	1.26, s	25.2 q	1.26, s	25.20 q	1.26, s
1″	29.3 t	3.26, d (7.4)	—	—	—	—		
2″	124.0 d	5.28, brt (7.4)	—	—	—	—	—	—
3″	132.7 s	—	—	—	—	—	—	—
4″	26.0 q	1.72, brs	—	—	—	—	—	—
5″	18.0 q	1.75, brs	—	—	—	—	—	—

a Chemical shift values were recorded using the solvent signal (CD_3_OD: *δ*_H_ 3.31, *δ*_C_ 49.00) as references.

Aspulvinone W (**2**): yellow solid (MeOH) [α]${}_{\text{D}}^{24}$‒32.8 (c 0.23, MeOH). UV (MeOH) λmax (log ε): 203 (4.35), 243 (3.98), 374 (4.15). Positive HR-ESI-MS: *m/z* measured 449.1963 [M + H]^+^, calcd for C_27_H_29_O_6_ [M + H]^+^ 449.1964. ^1^H NMR and ^13^C NMR spectroscopic data, see Table 1.

Aspulvinone X (**3**): yellow solid (MeOH) [α]${}_{\text{D}}^{24}$‒27.9 (c 0.26, MeOH). UV (MeOH) λmax (log ε): 203 (4.35), 242 (3.98), 376 (4.16). Positive HR-ESI-MS: m/z measured 449.1958 [M + H]^+^, calcd for C_27_H_29_O_6_ [M + H]^+^ 449.1964. ^1^H NMR and ^13^C NMR spectroscopic data, see Table 1.

### α-Glucosidase Inhibitory Assay

α-Glucosidase (EC:3.2.1.20, MAL12) from *Saccharomyces cerevisiae* was dissolved in 0.1 mol/L PBS solutions with a pH of 6.8, and diluted to be a 1.0 U/ml solution. The substrate p-nitrophenyl-β-D-glucopyranoside (pNPG) was dissolved in PBS to be a 1 mM solution. Acarbose and the compounds were dissolved in mehanol and further diluted to a series of concentrations from 0.1 μmol/L to 10 mmol/L. *In vitro* α-glucosidase inhibitory assay was performed according to a method described previously with some modification (Dan et al., 2019). Briefly, 20 μl of 1.0 U/ml enzyme solution and 10 μl of acarbose or compound solution, was mixed with 50 μl PBS solution in 96-well plate, and the mixed solution was incubated at 37°C for 10 min 20 μl of 1 mmol/L pNPG was subsequently added and further incubated at 37°C for 15 min, after which 100 μl of 1 M Na_2_CO_3_ solution was added to terminate the reaction. The absorbance of p-nitrophenol was monitored at 405 nm. All samples were analysed in triplicate, and acarbose was used as positive control. The negative control was performed by adding PBS instead of α-glucosidase, the blank was prepared by adding solvent without tested compounds. The inhibition rate was calculated as Eq. 1:

IR% =[(Ac-As)/Ac] × 100% (1)

where Ac represents the absorbance of control without sample solution, and As denotes the absorbance of sample.

### Enzymatic Kinetics of α-Glucosidase

pNPG with a concentration range of 100–4,000 μM and α-glucosidase were incubated with different concentrations of inhibitor for 10 min, respectively. 20 μl of 1.0 U/ml enzyme solutions were first mixed with 10 μl of different concentrations of inhibitors, then 50 μl PBS solutions were added, and the mixed solutions were incubated at 37°C for 10 min. Subsequently, 20 μl of pNPG solutions (1.25, 2.5, 5, 10 and 20 mM) were added, and the mixed solutions were further incubated at 37°C for 25 min, the absorbance of reaction solution was measured at 405 nm every 3 min. The kinetic parameters, Michaelis–Menten (K_m_) and maximum velocity (V_max_), were found using Lineweaver–Burk plots to check the mode of α-glucosidase inhibition for compounds **1** and **4**. The dissociation constants between inhibitor and enzyme (K_i_) were calculated from Dixon plots. Two inhibition constants, K_I_ or K_IS_, for inhibitor binding with either free or enzyme-substrate complex, were calculated from secondary plots of the slopes of the straight lines (V_max_/K_m_) or vertical intercept (1/V_max_) verse the concentration of inhibitors, respectively (Jenis et al., 2019).

### Fluorescence Quenching Analysis

α-Glucosidase (1 μM) was mixed with different concentrations of inhibitors (0–15 μM) at 20, 31, and 37°C, respectively, and the fluorescence spectra of mixed solutions were determined after equilibration for 5 min at an excitation wavelength of 250 nm. Both the excitation and emission slits were set at 10 nm. The quenching rate constant (K_q_), binding constant (K_a_), the number of the binding sites (*n*), and thermodynamic parameters enthalpy change (ΔH) and entropy change (ΔS) were calculated according to the Stern-Volmer Eq. 2 and the van’t Hoff Eqs 3–5, which were listed as follows (Xu et al., 2019):

$${\text{F}}_{\text{0}}/\text{F}{\;=\;1\;+\text{ K}}_{\text{sv}}\left[\text{Q}\right]{\;=\;1\;+\text{ K}}_{\text{q}}\tau_{\text{0}}\left[\text{Q}\right]$$ (2)

$$\text{log}\left(\left({\text{F}}_{\text{0}}-\text{F}\right)/\text{F}\right){\;=\text{ logK}}_{\text{a}}+\text{ nlogQ}$$ (3)

$${\text{lnK}}_{\text{a}}=\;\left(-1/\text{T}\left(\Delta \text{H/R}\right)\right)\;+\;\Delta \text{S}/\text{R}$$ (4)

ΔG = ΔH-TΔS (5)

Where F_0_ and F represent the fluorescence intensities in the absence or presence of inhibitor, [Q] denote the concentration of inhibitor, τ_0_ is the constant of the lifetime of fluorophore (10^–8^ s) and R is the gas constant of 8.31 J/(mol × K).

### Circular Dichroism Spectroscopy

The CD measurements were performed in a wavelength range of 190–250 nm at a speed of 60 nm/min. All measurements were carried out at 20°C using 1.0 mm path length quartz cuvette and sodium phosphate buffer (pH 6.8) was considered as a blank. The concentration of α-glucosidase was 1.25 μM, whereas the molar ratios of inhibitors (25 and 50 μM) to α-glucosidase were 20:1 and 40:1. All the results were expressed as ellipticity in mill degrees.

### Molecular Docking

Autodock Vina software was used in docking calculations to investigate the modes of glucosidase inhibition for individual aspulvinones. The 3D structures of aspulvinones were generated and then energetically minimized with MM_2_ force field to a minimum Root Mean Square (RMS) gradient of 0.005 using Chem3D Ultra 2017 (Version 17.0.0.206). The crystal structure of *Saccharomyces cerevisiae* isomaltase (PDB ID: 3A4A; Resolution 1.6 Å) was retrieved from protein Data Bank (www.rcsb.org/pdb/), and was prepared by removing the water molecules and original inhibitors. The ligand and protein pdb files were prepared and grid box formation was accomplished using AutoDock Tools. AutoGrid was used in order to prepare the grid map using a grid box. The dimension of the grid size was set to 50 × 50 × 50, and the grid box center was designated in coordinates x = 21.285, y = −0.64, and z = 18.475. Nine output poses were generated and evaluated by their calculated free energy of binding. The best pose of each ligand was determined by its Affinity score (kcal/mol), which was visualized by Discovery Studio Visualizer v21.1.0.20298 (Accelrys, San Diego, United States) to analyze the interactions between the target enzyme and the inhibitor.

### Oral Disaccharide Tolerance Test

Female C57BL/6J mice, 6 weeks old, weighting 16–20 g, were obtained from the Skbex Biotechenology Company Limited (Henan, China). The animals were housed in Experimental Animal Center of Zhoukou Normal University under 12 h light-dark cycle at controlled temperature (22 ± 1°C), and provided with a standard pellet diet and water ad libitum. The mice were adapted to diet and general conditions of vivarium for 1 week before the experiment. After an 16 h fasting, the mice were divided into three groups randomly (eight mice each group). Sucrose or maltose, as well as the inhibitors (compound **4** and acarbose), was dissolved in 0.5% sodium carboxymethyl cellulose (CMC–Na) solution. Compound **4** was tested at dose of 25 mg/kg, whereas acarbose was evaluated at dose of 50 mg/kg of body weight (BW). Mice were fasted 16 h and then intragastrically (i.g.) administrated the inhibitors, 15 min later, 2 g/kg BW of sucrose or 2 g/kg BW of maltose solution was given i.g. to the animals. Blood samples were taken from the tail vein at 0, 30, 60, and 120 min after sucrose or maltose loading, and blood glucose was measured with Accu-Chek Active glucometer (Roche, Germany). Area under the curve (AUC) over 0–120 min was calculated by the trapezoidal method. All animals were cared under the frame of the China Council on Animal Care and all procedures were approved by the Health Sciences Animal Welfare Committee of Zhoukou Normal University.

## Results and Discussion

### Isolation and Structure Determination of Aspulvinones

The mutant ASM-1 was obtained with different colonial morphologies with the parent strain ML-44 (Supplementary Figure S1), through treatment of ML-44 spores with 2% (v/v) DES under ultrasonic assistance for 1 h, and its EtOAc extract HPLC profile showed a series of chromatographic peaks of newly produced secondary metabolites with unique UV absorption spectra comparing to the wild strain ML-44 (Supplementary Figure S2). This mutant was deposited at the China General Microbiological Culture Collection Center under the accession number CGMCC No. 22417. In order to clarify the newly produced secondary metabolites, large scale fermentation of mutant ASM-1 and HPLC-guided separation were performed (Supplementary Figure S3). The EtOAc extract of the mutant ASM-1 was separated by silica gel column chromatography and repeated preparative reverse phase HPLC separation under HPLC-PDAD-UV monitoring, resulted in the isolation of aspulvinones **1**–**6**. Compound **4** was determined to be aspulvinone H by comparison its MS and NMR data with that of literature (Nagia et al., 2012). Compound **6** was identified as aspulvinone R, which was recently isolated from a marine sponge-associated fungus *A. flavipes* KUFA1152 as the first example of triprenylated aspulvinones (Machado et al., 2021).

**FIGURE 2:**
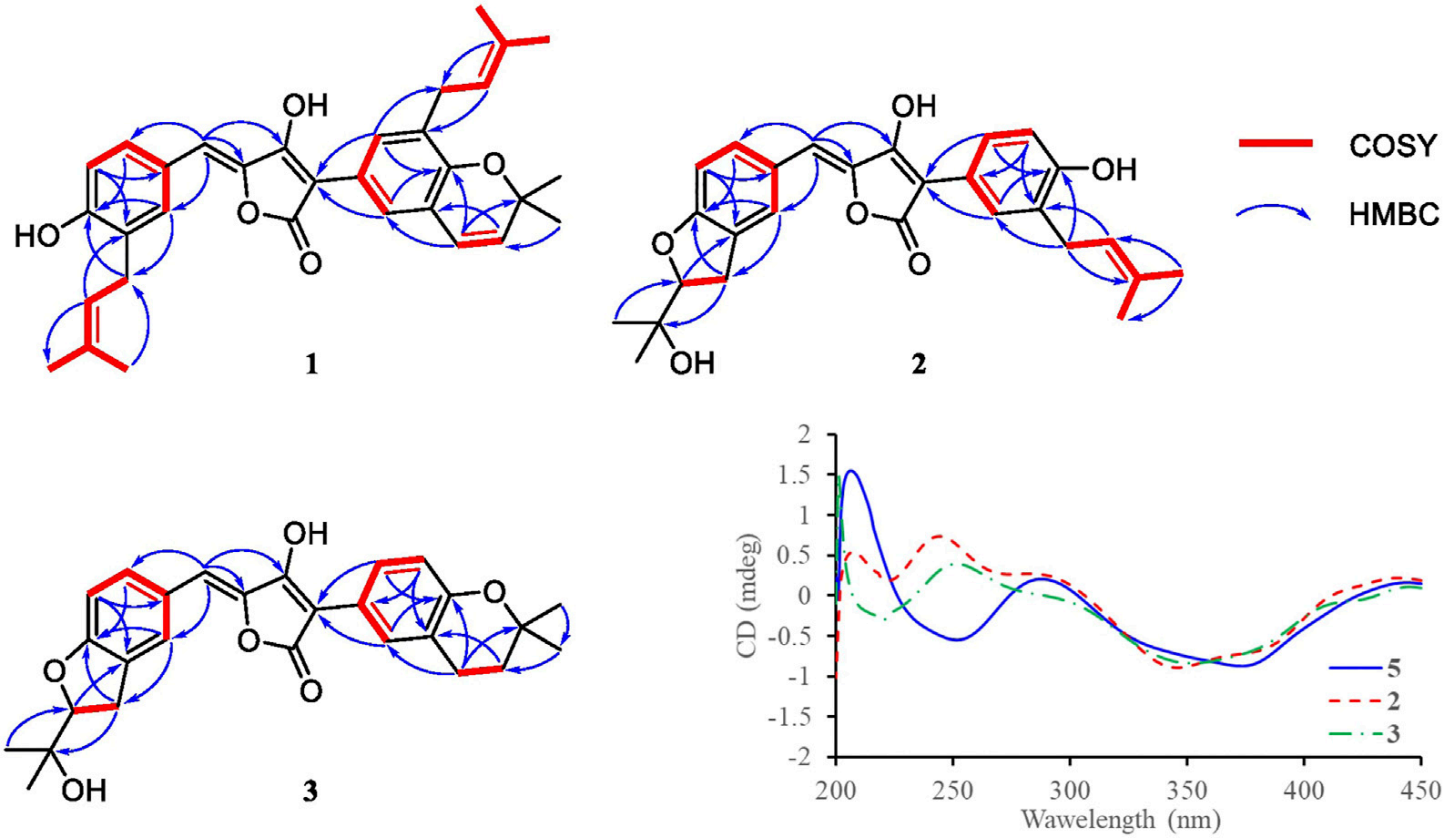
Key COSY and HMBC correlations of compounds **1**–**3**, and CD spectra of compound **2**, **3**, and **5**.

Compound **1** was obtained as yellow solid. The molecular formula was determined to be C_32_H_34_O_5_ on the basis of a HRESIMS peak at m/z 499.2480 [M + H]^+^ (calcd. 499.2484), indicating 16 degrees of unsaturation. The illustration of ^13^C NMR, DEPT, and HSQC spectra came up with 32 resonances, which were indicative of one ketone carbonyl, twelve *sp* ^*2*^ and one *sp* ^*3*^ quaternary carbons, ten *sp* ^*2*^ methine, two *sp* ^*3*^ methylene, six methyl groups in **1**. The remaining unsaturation was thus attributed to four rings. The ^1^H NMR spectroscopic data of **1** (Table 1) showed a series of protons signals, and were affiliated to relevant carbons via HSQC analysis. The detailed 1D (Table 1) and 2D (Figure 2) NMR analyses of **1** indicated the same pulvinone nucleus as **4** and **6**. The ^1^H NMR signals δ6.36 (d, J = 9.7 Hz, H-18) and δ5.68 (d, J = 9.7 Hz, H-19) indicated that one of the prenyl occurred cyclization, which was verified by the HMBC correlations between the above two protons with related carbons (Figure 2). In addition, there were two linear prenyl groups (C-1′ to C-5′, and C-1″ to C-5″) in the molecular of **1** according the NMR data analysis. Thus, compound **1** should also be a triprenylated pulvinone. Subsequently, the HMBC correlations between H-18 to C-13 and C-15, H-1″ to C-17 and H-2″ to C-16, H-19 to C-14, H-13 and H-17 to C-2, indicated that a 1,3,4,5-tetrasubstituted benzene ring bound to the γ-butenolide core directly at C-2. Thus, the another one prenylated benzene ring linked to the core via C-5, which was also confirmed by relative HMBC signals. At this stage, the planar structure has been constructed as **1**. The relatively small chemical shifts of C-2 (δ_C_ 102.2) and C-5 (δ_C_ 109.5) established the Z geometry of the ∆_4,5_-double bond (Campbell et al., 1985), which was the same as that of compound **6**. Since **1** has never been reported previously, it was named as aspulvinone V in the order of the names for this series of prenylated pulvinones from *A. terreus*.

Compound **2** was obtained as yellow solid. Its molecular formula was established as C_27_H_28_O_6_ on the basis of a HRESIMS peak at *m/z* 449.1963 [M + H]^+^ (calcd. 449.1964), which indicated 14 degrees of unsaturation and 16 amu more than compound **4** (C_27_H_28_O_5_). The ^1^H and ^13^C NMR data (Table 1) indicated that the structure of **2** is very similar to compound **4**. The most significant differences in the NMR data exist in the high-field shift effect of the signal at C-1′ (δ_H_ 3.20, 3.15, δ_C_ 31.2), and the presence of a O-substituted *sp* ^*3*^ methine instead of a *sp* ^*2*^ methine at C-2′ (δ_H_ 4.59, δ_C_ 91.1), a O-substituted *sp* ^*3*^ quaternary carbon instead of a *sp* ^*2*^ one at C-3′ (δ_C_ 72.4). These data indicated that compound **2** bears a dihydrofuran ring fused to the benzene ring, as opposed to the linear prenyl present in compound **4**. This structure was further supported by COSY, HSQC, and HMBC spectra. COSY correlations between H_2_-1′ and H-2′, as well supported the presence of one dihydrofuran ring systems. HMBC correlation from H_2_-1′ to C-8 and C-9, H-10 to C-8, H-7, and H-11 to C-9 confirmed the presence of a dihydrofuran fused to one of the benzene rings. Furthermore, HMBC signals of H-5 to C-7 and C-11 indicated the dihydrofuran fused benzene ring was linked to C-5.

Compound **3** was isolated as yellow solid with the same molecular formula as **2** base on HRESIMS peak at *m/z* 449.1958 [M + H]^+^ (calcd. 449.1964). The ^1^H and ^13^C NMR data of **3** (Table 1) were similar to those of **2** except for the high-field shift effect of the signal at C-18 (δ_H_ 2.81, δ_C_ 23.4), the presence of a *sp* ^*3*^ methylene instead of a *sp* ^*2*^ methine at C-19 (δ_H_ 1.82, δ_C_ 33.8), and a O-substituted *sp* ^*3*^ quaternary carbon instead of a *sp* ^*2*^ one at C-20 (δ_C_ 75.5). These data indicated that compound **3** bears a tetrahydropyran ring fused to the benzene ring, instead of the linear prenyl present in compound **2**. The location of furan ring and pyran ring were confirmed through HMBC analysis (Figure 2). In addition, both **2** and **3** have the Z geometry for the ∆_4,5_-double bond according to ^13^C NMR data. The transisomer of compound **3** (*trans*-**3**) has been reported previously, which was determined the absolute configuration as *R* by the ECD calculations (Sun et al., 2018). The CD spectra of **2** and **3** was tested, and the negative cotton effect at 350 nm and general spectrum shapes were both consistent with *trans*-**3** (Figure 2), indicating the *R* configuration at C-2′ for compounds **2** and **3**.

**FIGURE 3:**
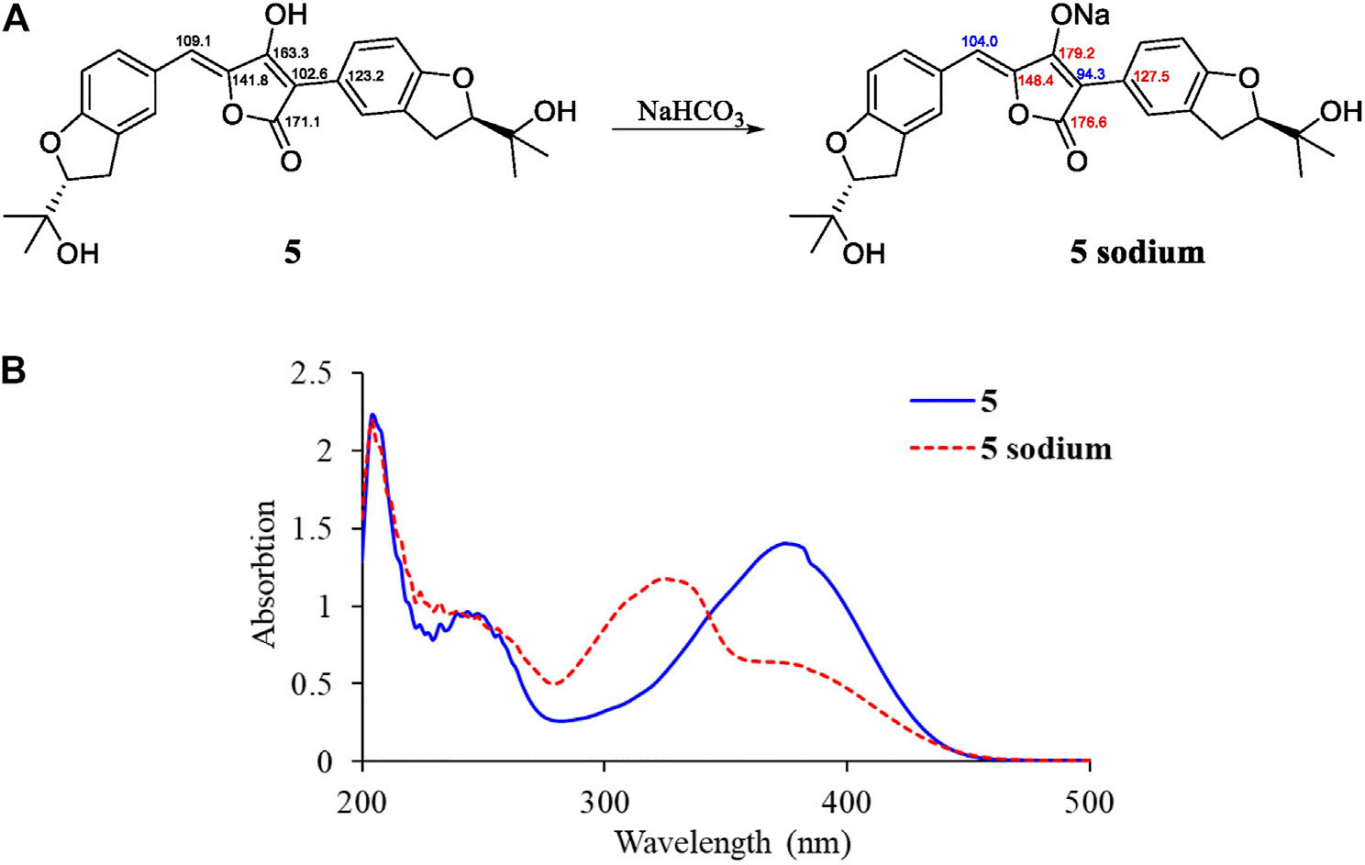
Spectral comparisons between **5** and **5** sodium. **(A)** ^13^C-NMR data differences; **(B)** UV spectra of **5** and **5** sodium.

By illustration of 1D and 2D NMR data, compound **5** was deduced to have the same structure with aspulvinone J-CR, whereas there exists considerable discrepancy between our ^13^C NMR data and that of the literature (Cruz et al., 2011), especially for C2–C5 and C12. Taking consideration of the relatively strong acidity of 4-OH (predicted pK_a_ value of 4.50 ± 1.00 calculated by Advanced Chemistry Development (ACD/Labs) Software V11.02), it is presumed that pulvinone derivatives may be dissociative in relative high pH solutions, which would cause changes in NMR and UV spectral characteristics. Based on this assumption, **5** sodium was prepared by adding NaHCO_3_ aqueous solution to **5** methanol solution in molar ratio 1:1. The ^13^C NMR data of **5** sodium exhibited significant difference with **5** (Figure 3A), while it is identical with the literature data. Therefore, the NMR data for aspulvinone J-CR and other analogs in the literature should be for their sodium. In addition, the UV spectra of the **5** and its sodium represented different maximum absorption peaks both in number and intensity (Figure 3B), **5** has one absorption peak at 376 nm, while **5** sodium exhibited two absorption peaks at 327 and 376 nm, and the latter with a relatively low absorbance exists as a shoulder peak of the former.

### α-Glucosidase Inhibitory Activities

All compounds **1**–**6** showed potent inhibitions towards α-glucosidase with IC_50_s ranging from 2.2 to 44.3 μM (Table 2). It was reported that the transisomer of compound **3** inhibited α-glucosidase with IC_50_ of 24.8 μM (Sun et al., 2018). The inhibitory potencies varied with the modification of benzene rings. In term of diprenylated aspulvinones, compound **4** (IC_50_ 4.6 μM) is appropriate ten times more potent than **2**, **3** and its transisomer, and **5**, indicating that the linear prenyl is significant to the α-glucosidase inhibitory activity, while the configuration of ∆4,5-double bond has little influence. However, Aspulvinone E (IC_50_ 2.70 μM) was reported with higher inhibitory activity than its transisomer, isoaspulvinone E (IC_50_ 8.92 μM), and the ∆4,5-double bond stereochemistry significantly affected the inhibition activity to α-glucosidase for non-prenylated pulvinones (Dewi et al., 2014). Therefore, we presumed that non-prenylated and prenylated pulvinones had different binding modes with α-glucosidase. On the other hand, both of compound **1** (IC_50_ 2.2 μM) and **4** were more potent than **6** (IC_50_ 10.8 μM). It was speculated that the fork-like structure of two linear prenyl groups has steric hindrance effect hampering the binding of **6** with enzyme.

**TABLE 2:** α-Glucosidase inhibitory activity of aspulvinones **1**–**6**.

Compounds	IC50 (µM)^a^	K_i_ (µM)^b^	K_I_ (µM)	K_IS_ (µM)	Inhibition mode
**1**	2.2 ± 0.4	6.60	3.15	8.23	mixed-type
**2**	32.0 ± 5.8	NT^c^	NT	NT	NT
**3**	38.6 ± 5.2	NT	NT	NT	NT
**4**	4.6 ± 1.3	6.58	4.70	6.62	mixed-type
**5**	44.3 ± 8.9	NT	NT	NT	NT
**6**	10.8 ± 2.3	NT	NT	NT	NT
Acarbose^d^	17.2 ± 1.8	NT	NT	NT	NT

a Sample concentration which led to 50% enzyme activity loss.

b Ki is the inhibition constant.

c NT is not tested.

d Acarbose is used as a positive control.

**FIGURE 4:**
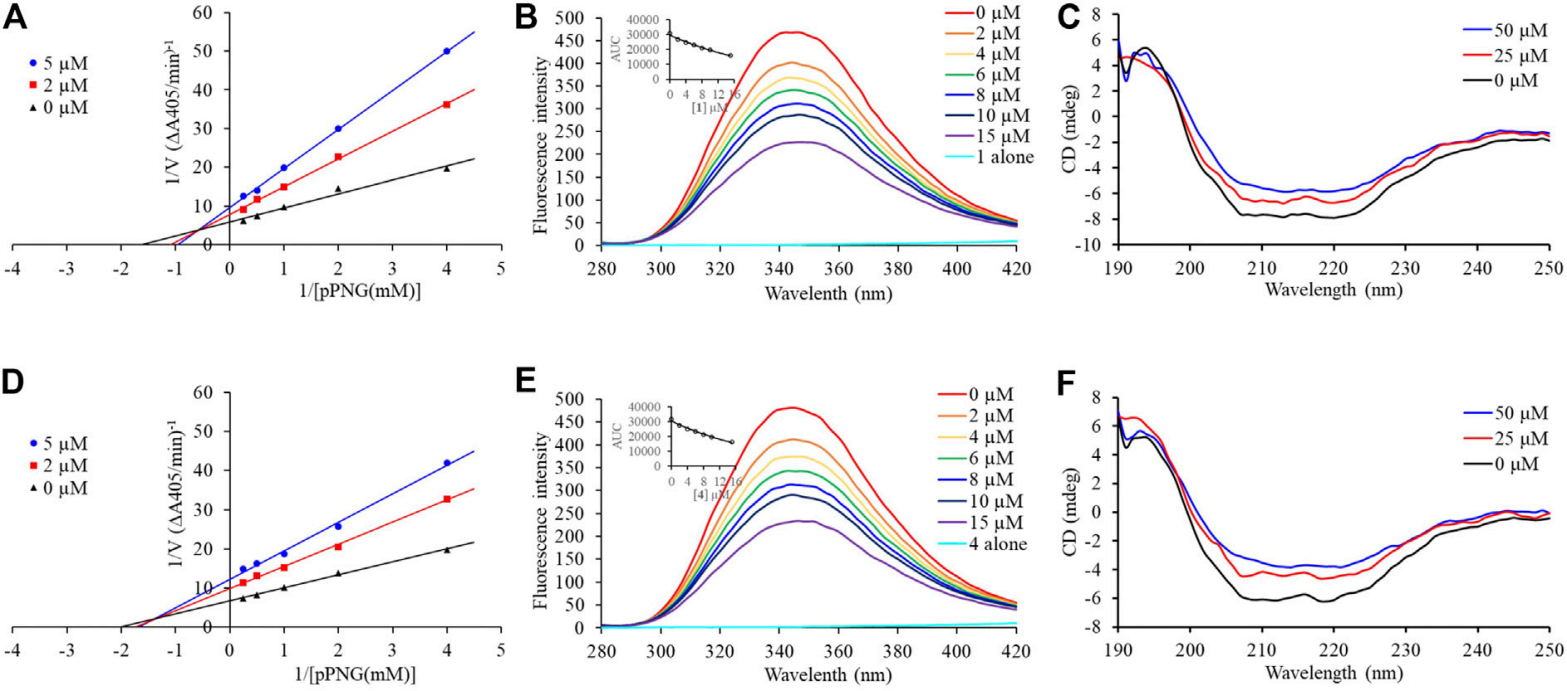
Inhibition mechanism of **1** and **4** against α-glucosidase. Lineweaver–Burk plot of **1 (A)** and **4 (D)**. Fluorescence spectra of α-glucosidase with **1 (B)** and **4 (E)**. CD spectra of α-glucosidase with **1 (C)** and **4 (F)**.

Compounds **1** and **4**, the most potent inhibitors, were selected for enzyme kinetic studies to elucidate the inhibition mode. In the Lineweaver–Burk double-reciprocal plots, as shown in Figure 4, the plots of 1/V versus 1/[S] give a group of straight lines with different slopes that intersect at the second quadrant for both of **1** and **4**, suggesting that both of them are mixed-type inhibitors (Dan et al., 2019). Therefore, both compounds could bind to free enzyme (EI), and interfere with the formation of the α-glucosidase-pNPG (ES) intermediate through forming an α-glucosidase-pNPG-inhibitor (ESI) complex (Wikul et al., 2012; Wu et al., 2014). The inhibition constant for the inhibitor binding with free enzyme (K_I_) was determined by a plot of the slope (K_m_/V_m_) versus the inhibitor concentration, and the inhibition constant for the inhibitor binding with enzyme–substrate complex (K_IS_) was obtained from the vertical intercept (1/V_m_) versus the inhibitor concentration (Supplementary Figure S5) (Sheng et al., 2018). The results are shown in Table 2: the K_I_ values of both **1** and **4** are smaller than their K_IS_ values, which suggest that them have higher affinity with the free enzyme than with the enzyme-substrate complex.

### Fluorescence Quenching Mechanism and Binding Characterizations

Subsequently, the interaction between the inhibitors and α-glucosidase was investigated by fluorescence spectroscopy and circular dichroism (CD) spectroscopy. As shown in Figure 4, with the increased concentrations of **1** and **4**, the intrinsic fluorescence intensity of α-glucosidase decreased gradually, indicating that the inhibitors interacted with α-glucosidase and then quenched its intrinsic fluorescence. Comparing to the maximum scattering collision quenching constant of the biomacromolecule (2 × 10^10^ L/mol/s), the quenching rate constants (K_q_) were much larger, demonstrating that the fluorescence quenching process was static quenching predominantly (Table 3). The number of binding sites (n) were all close to one at the three incubation temperatures, indicating that both **1** and **4** interact with α-glucosidase at only one binding site. The binding constants (K_a_) at the three temperatures were in the order of 10^5^ and 10^4^ L/mol for **1** and **4**, respectively, indicating that there were high binding affinities existed in the complex of α-glucosidase with the both compounds, especially as for **1**. In addition, the thermodynamic parameters (∆S, ∆H and ∆G) were calculated, showing that ∆H and ∆S were positive, while ∆G was negative (Table 3). The binding process could be defined to be thermodynamically favorable and spontaneous, which was driven mainly by a hydrophobic force (Ross and Subramanian, 1981). In the CD spectroscopy analysis, after the addition of 25 and 50 mM of **1** or **4**, the absorption of the two negative peaks at 209 and 222 nm decreased, which demonstrated a loss of the α-helix structure (Figure 4) (Xu et al., 2019). In addition, with an increase in molar ratios of inhibitors to α-glucosidase (from 20:1 to 40:1), the loss of the α-helix structure increased, associating with the decreased α-glucosidase activity. The transformation from the α-helix to other conformations in the presence of inhibitors indicated a partial unfolding of the α-glucosidase structure, causing alterations of the secondary structure of α-glucosidase, and thereby some hydrogen bonding networks might be destroyed. These alterations may prevent the binding of the substrate to α-glucosidase or hamper the formation of an active center, eventually resulted in the dysfunction of the enzyme (Xu et al., 2019).

**TABLE 3:** Quenching constants (Ksv), binding constants (Ka), number of binding sites (*n*), and thermodynamic parameters for the α-glucosidase-inhibitor system.

Inhibitor	T (^o^C)	K_sv_ (× 10^4^ L/mol)/K_q_ (× 10^12^ L/mol/s)	K_a_ (× 10^4^ L/mol)	n	*∆*G (kJ/mol)	*∆*H (kJ/mol)	*∆*S (J/mol/K)
**1**	20	5.35	11.0	1.04	−14.8	31.2	148.2
	31	7.87	30.6	1.12	−13.9		
	37	5.81	56.0	1.23	−12.3		
**4**	20	5.69	2.9	0.92	−14.0	60.4	240.1
	31	5.53	7.9	1.04	−12.5		
	37	6.02	23.2	1.13	−9.9		

### Molecular Docking

**FIGURE 5:**
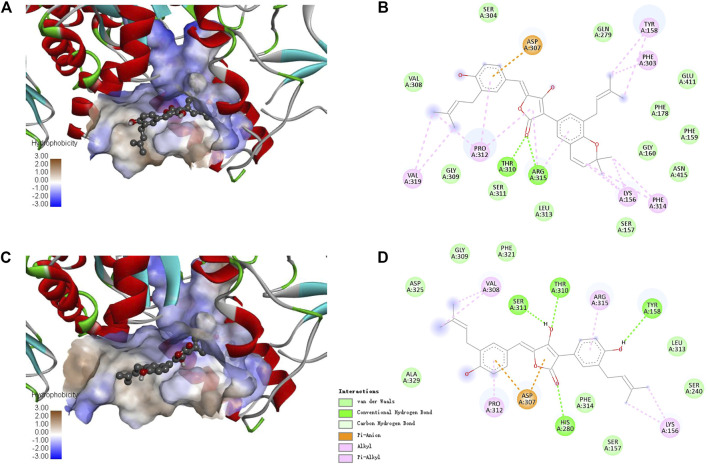
Docking binding model of inhibitors with *Saccharomyces cerevisiae* isomaltase (3A4A). Predicted dock conformation of the isomaltase to inhibitors **1 (A)** and **4 (C)**; 2D interaction diagrams between 3A4A and inhibitors **1 (B)** and **4 (D)**.

Since there is no crystal structure for the commercially available Saccharomyces cerevisiae α-glucosidase for preparing the protein for docking, the constructed homology models based on the isomaltases or itself were often used to perform the molecular docking (Xu et al., 2019; Khosravi et al., 2020). In this study, the crystal structure of isomaltase from *Saccharomyces cerevisiae* (PDB ID: 3A4A; Resolution 1.6 Å) was adopted for silico docking of to confirm the interaction. Compounds **1** and **4** exhibits a strong binding affinity with the protein by the low binding energy of −10.9 and −9.6 kcal/mol, respectively. As shown in Figure 5, both compounds could bond at the gate of the hydrophobic pocket, and partially inserted into the binding pocket. In this bonding mode, the ligands could hamper the substrate loading into the catalytic pocket in EI complex formation, or cause structural modification of α-glucosidase leading to the dysfunction in ESI complex formation (Kim et al., 2005). According to the molecular docking results, the binding pocket involves the amino acid residues Asp307, Pro312, Tyr158, Thr310, Arg315, and Lys156 for the both inhibitors, and additional Phe303, Phe314 and Val319 for **1**, whereas additional Val308, Ser311, and His280 for **4**. There were the hydrogen bond interactions between the carbonyl group of **1** with Thr310 and Arg315 (the distance: 2.26 and 2.62 Å), and the rest interactions were all hydrophobic effect including alkyl, Pi-alkyl and Pi-anion (Figure 5C). In comparison, there are relatively more hydrogen bonds and less hydrophobic interaction for **4** (Figure 5D), indicating the different binding force compositions between the two inhibitors.

### Effect of Compound 4 on Postprandial Hyperglycemia *in vivo*

**FIGURE 6:**
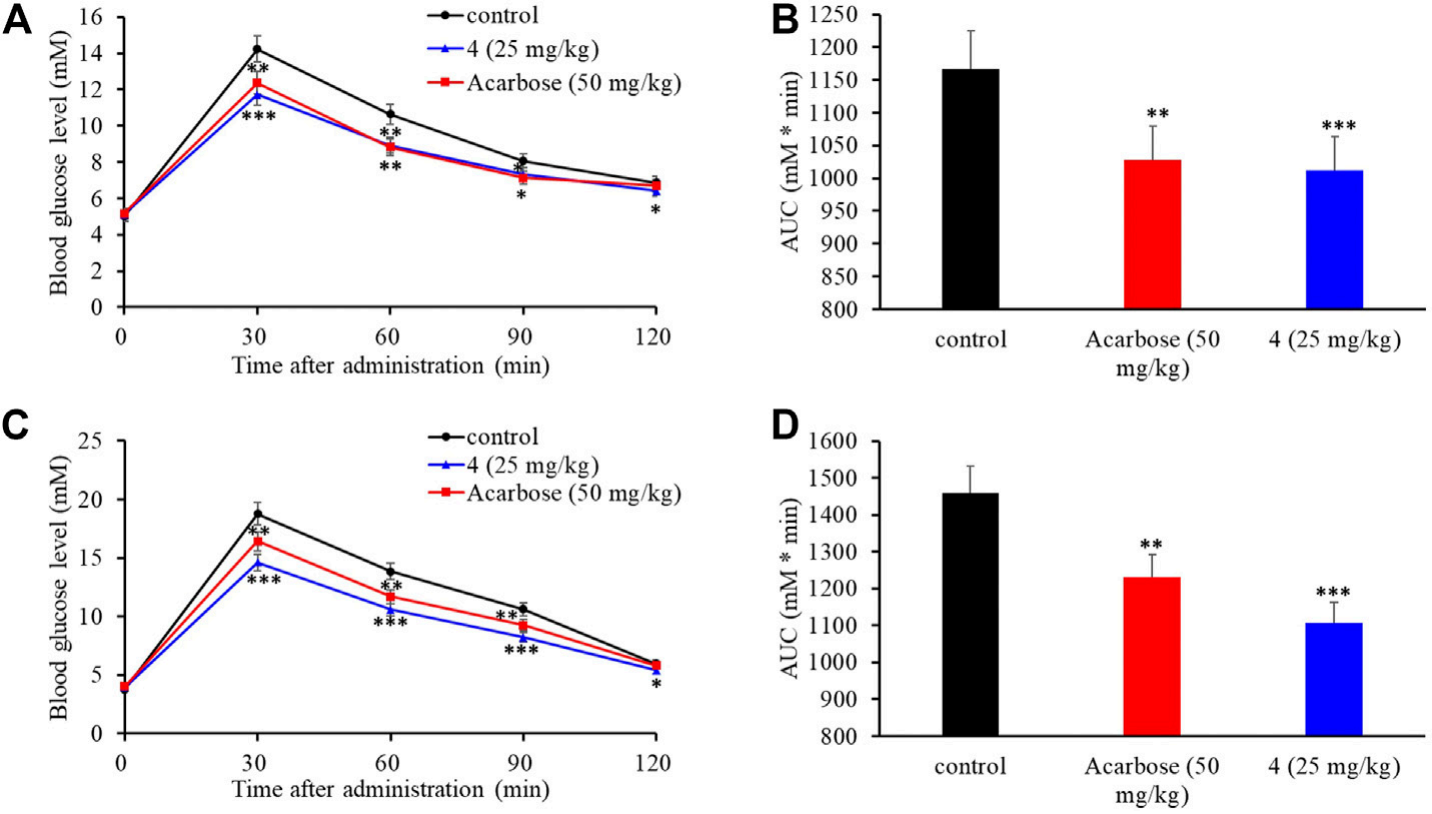
Effects of acarbose and **4** on postprandial blood glucose levels in female C57BL/6J mice. **(A)** Blood glucose concentrations after oral administration of **4** and sucrose. **(B)** Area under the curve (AUC) after oral administration of **4** and sucrose for 2 h. **(C)** Blood glucose concentrations after an oral administration of **4** and maltose. **(D)** Area under the curve (AUC) after oral administration of **4** and maltose for 2 h. Each value represents the mean standard deviation (*n* = 8). Asterisks indicate a significant difference (*p < 0.05, **p < 0.01, and ***p < 0.001) compared with the control group.

The intestinal α-glucosidase inhibitory activity *in vivo* was evaluated by oral sucrose and oral maltose tolerance tests in female C57BL/6J mice. Acarbose (50 mg/kg BW) was used as a positive control, and compound **4** was chosen for its potent inhibitory activity and high yield. In the oral sucrose tolerance test (Figure 6A), after oral administration of sucrose (2 g/kg of BW), the blood glucose level rapidly increased from 5.00 ± 0.07 mM to a maximum of 14.24 ± 0.45 mM in 30 min, and then recovered to the pretreatment level at 120 min. In the treatment group, **4** significantly suppressed the blood glucose rise at 30 and 60 min comparing to that of the negative control group, and led to 13.2% decrease of the AUC at a dose of 25 mg/kg BW comparable to that of acarbose (11.8% decrease) at dose of 50 mg/kg BW (Figure 6B). Similarly, in the sucrose tolerance test, compound **4** treatment resulted in a significant decrease in the postprandial blood glucose peak versus the negative control group (Figure 6C) and he AUC for postprandial plasma glucose was reduced by 19.7% in 2 h after **4** administration, which was more potent than acarbose (16.2%) (Figure 6D). These results strongly confirmed that **4** could alleviate the postprandial hyperglycemia through inhibiting intestinal α-glucosidase. Therefore, natural aspulvinones can be regarded as potential candidate for hpyerglycemic agents.

## Conclusion

In summary, we conducted the DES mutagenesis on the marine-derived *A. terreus* ML-44, and a mutant strain ASM-1 was obtained by morphological and HPLC analyses. Six aspulvinone secondary metabolites were isolated from the ASM-1 culture, including three new ones. Their structures including the absolute configurations were elucidated by various spectroscopic methods and ECD comparison. All compounds were evaluated for α-glucosidase inhibitory activity with acarbose as positive control. Among them, compounds **1** and **4** exhibited potent α-glucosidase inhibitory activities with IC_50_ values of 2.2 and 4.6 µM in mixed-type manners. The thermodynamic and molecular docking studies recognized the interaction between inhibitors and α-glucosidase was spontaneous exothermic reaction driven mainly by hydrophobic forces. Furthermore, **4** significantly suppressed the increases in postprandial blood glucose levels in the C57BL/6J mice more potently than acarbose at a smaller dosage. The results suggested that aspulvinones could be promising candidates for further pharmacologic research. In addition, the mechanism of the mutagenesis of the strain ASM-1 from strain ML-44 deserve further investigation through genome and transcriptome analyses, which may make contribution to understanding the metabolic regulation of aspulvinones biosynthesis.

## Data Availability

The original contributions presented in the study are included in the article/Supplementary Material, further inquiries can be directed to the corresponding authors.
