# Supplementary material for: Aspulvinones Suppress Postprandial Hyperglycemia as Potent α-Glucosidase Inhibitors From Aspergillus terreus ASM-1
Source: Front Chem. 2021 Aug 17;9:736070. doi: 10.3389/fchem.2021.736070 (PMC8416056; doi:10.3389/fchem.2021.736070)
Supplement: Supplementary file 1 [file DataSheet1.PDF]

frontiers

Supplementary Material

Aspulvinones suppress postprandial hyperglycemia as potent α-glucosidase inhibitors from *Aspergillus terreus* ASM-1

Changjing Wu[1,2,3†], Xiang Cui[1†], Luzhen Sun[1], Jiajia Lu[1], Feng Li[1], Minghui Song[1], Yunxia Zhang[1], Xinqi Hao[2], Congkui Tian[3*], Maoping Song[2*] and Xiaomeng Liu[1,4*]

Affiliation

[1]College of Life Sciences and Agronomy, Zhoukou Normal University, Zhoukou, China

[2]College of Chemistry and Molecular Engineering, Zhengzhou University, Zhengzhou, China

[3]Wuling Mountain Institute of Natural Medicine, Hubei Minzu University, Enshi, China

[4]College of Public Health, Xinxiang Medical University, Xinxiang, China

*** Correspondence:**
Xiaomeng Liu
lxmxm_99@126.com

Maoping Song
mpsong@zzu.edu.cn

Congkui Tian
pkutianck@163.com

†These authors contributed equally to this work

List of Supplementary Materials

Figure S1. Colonial morphologies of Aspergillus terreus ML-44 and its mutant strains 1
Figure S2. Comparative HPLC-DAD-UV analysis between the EtOAc extracts 1
Figure S3. HPLC-PDAD-UV analysis for ASM-1 large-scale fermentation and its fractions..........2
Figure S4. Chemical stability tests for compound **4** in acidic solution or under ultraviolet light 3
Figure S5. Plot of the slope (Km/Vm) versus the inhibitor concentration for compounds **1** (a) and **4** (b), and plot of vertical intercept (1/Vm) versus the inhibitor concentration for **1** (c) and **4** (d).........3
Figure S6. Full view of dock conformation of the isomaltase (3A4A) to inhibitors **1** (a) or **4** (b).....3
Figure S7. HRESIMS data of compound **1**..4
Figure S8. ^{1}H-NMR spectrum of compound **1** in CD_3OD..4
Figure S9. ^{13}C-NMR and DEPT-135 spectra of compound **1** in CD_3OD..........................5
Figure S10. HSQC spectrum of compound **1** in CD_3OD...5
Figure S11. ^{1}H-^{1}H COSY spectrum of compound **1** in CD_3OD.......................................6
Figure S12. HMBC spectrum of compound **1** in CD_3OD...6
Figure S13. HRESIMS data of compound **2**..7
Figure S14. ^{1}H-NMR spectrum of compound **2** in CD_3OD...7
Figure S15. ^{13}C-NMR and DEPT-135 spectra of compound **2** in CD_3OD........................8
Figure S16. HSQC spectrum of compound **2** in CD_3OD...8
Figure S17. ^{1}H-^{1}H COSY spectrum of compound **2** in CD_3OD.......................................9
Figure S18. HMBC spectrum of compound **2** in CD_3OD...9
Figure S19. HRESIMS data of compound **3**..10
Figure S20. ^{1}H-NMR spectrum of compound **3** in CD_3OD...10
Figure S21. ^{13}C-NMR and DEPT-135 spectra of compound **3** in CD_3OD......................11
Figure S22. HSQC spectrum of compound **3** in CD_3OD...11
Figure S23. ^{1}H-^{1}H COSY spectrum of compound **3** in CD_3OD.....................................12
Figure S24. HMBC spectrum of compound **3** in CD_3OD...12
Figure S25. HRESIMS data of compound **5**..13
Figure S27. DEPT-135 spectrum of compound **5** in CD_3OD..14
Figure S29. HSQC spectrum of compound **5** in CD_3OD...15
Figure S30. ^{1}H-^{1}H COSY spectrum of compound **5** in CD_3OD.....................................15
Figure S31. HMBC spectrum of compound **5** in CD_3OD...16

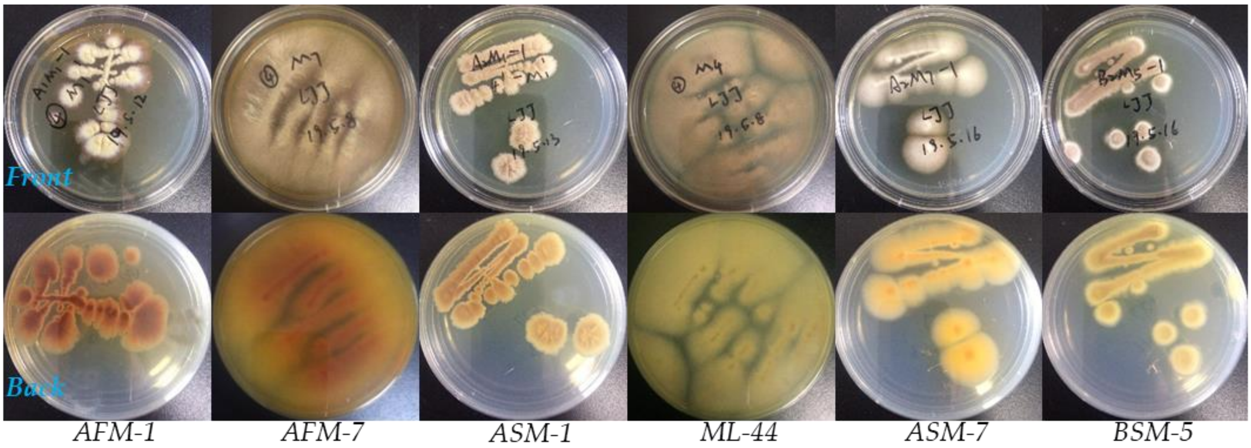

Figure S1. Colonial morphologies of Aspergillus terreus ML-44 and its mutant strains.

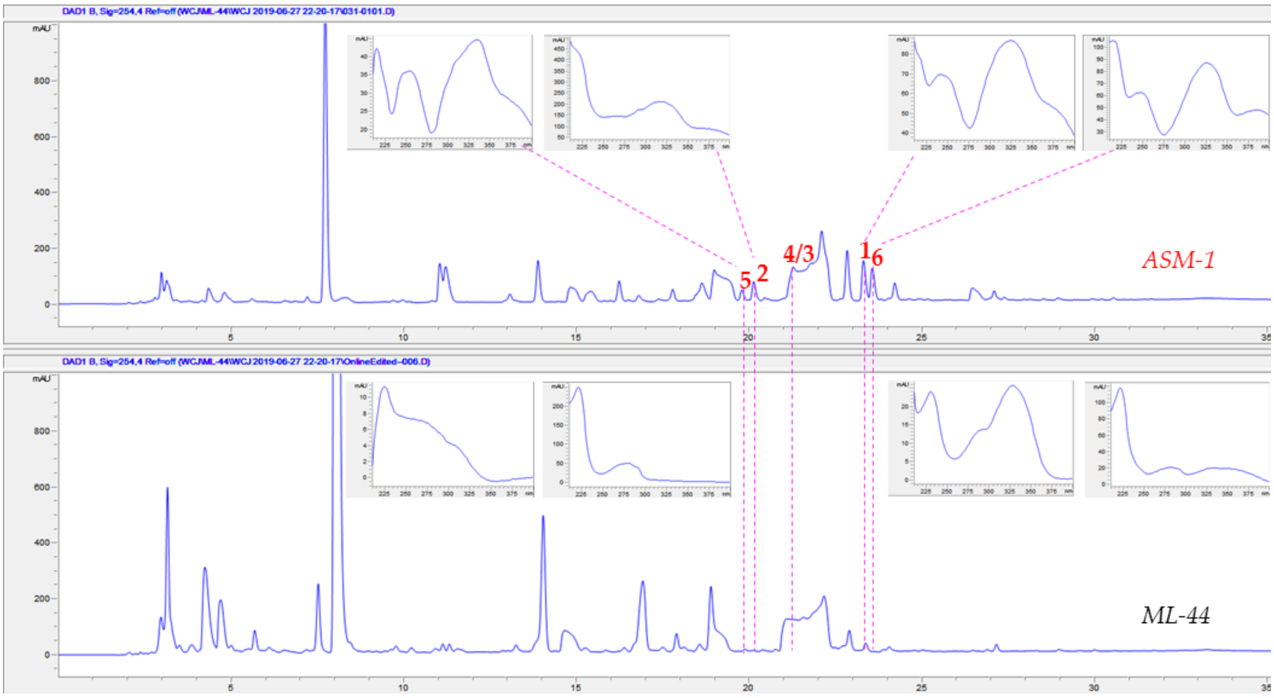

Figure S2. Comparative HPLC-DAD-UV analysis between the EtOAc extracts of strain ML-44 and ASM-1 cultures.

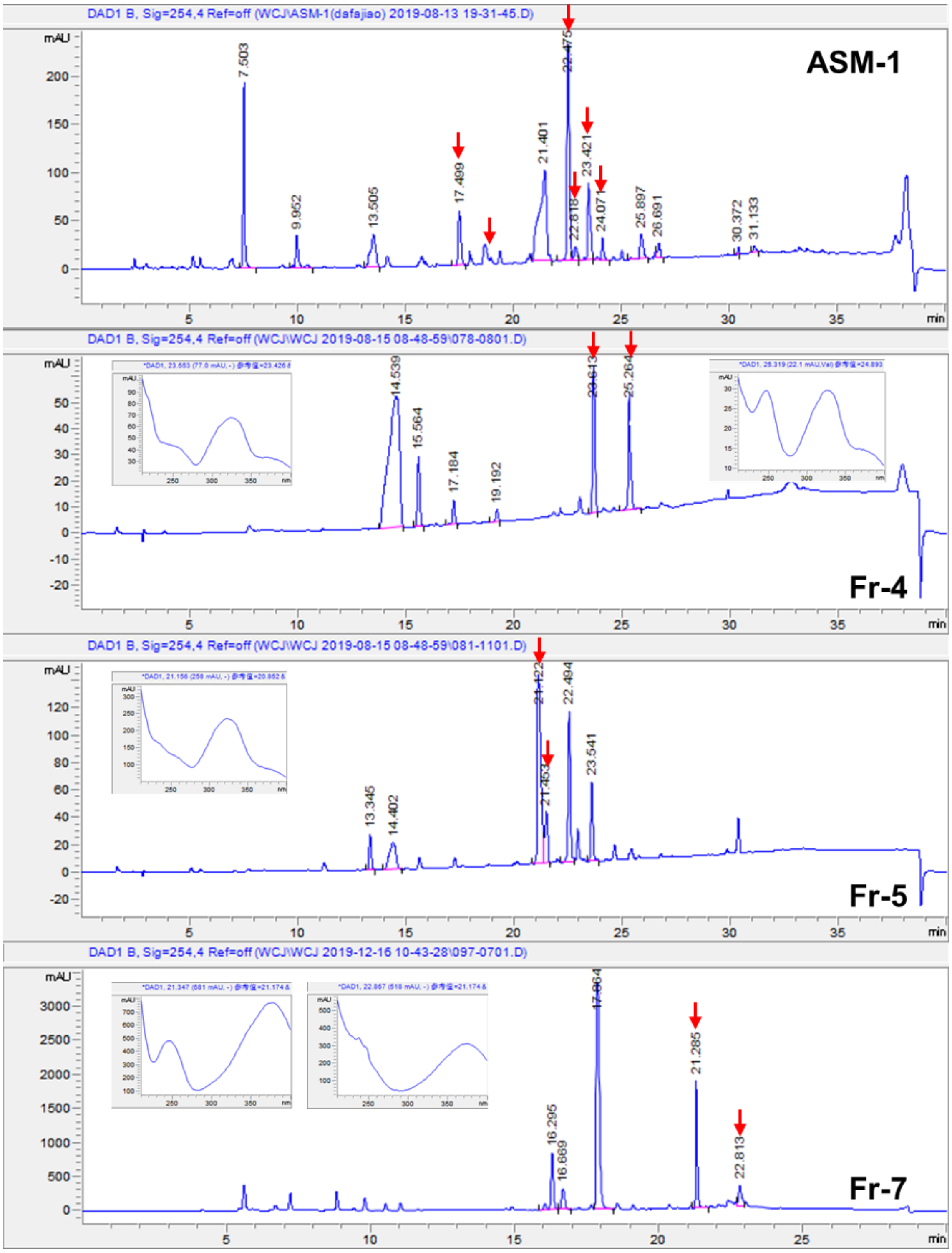

Figure S3. HPLC-PDAD-UV analysis for ASM-1 large-scale fermentation and its fractions. HPLC conditions: MeOH-H_2O linear gradient (20%→100% MeOH in 30 min followed by 5 min with isocratic 100% MeOH) mobile phase (1 mL/min flow rate) for ASM-1, Fr-4, and Fr-5; MeOH-H_2O (0.1%HCl) linear gradient (20%→100% MeOH in 20 min followed by 5 min with isocratic 100% MeOH) mobile phase (1 mL/min flow rate) for Fr-7.

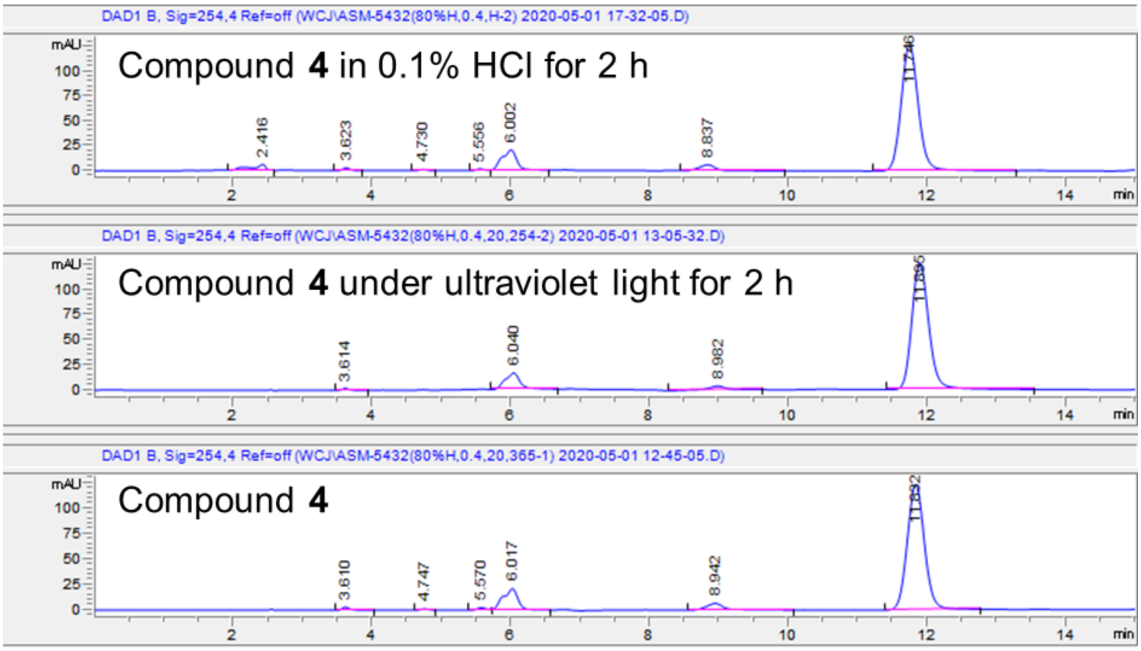

Figure S4. Chemical stability tests for compound **4** in acidic solution or under ultraviolet light. HPLC conditions: MeOH-H_2O (0.1%HCl) 80:20, 1 mL/min.

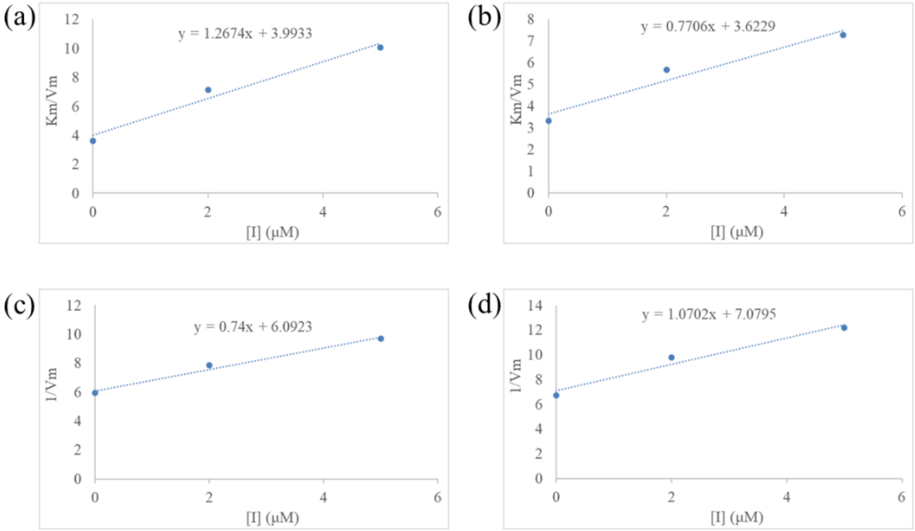

Figure S5. Plot of the slope (Km/Vm) versus the inhibitor concentration for compounds **1** (a) and **4** (b), and plot of vertical intercept (1/Vm) versus the inhibitor concentration for **1** (c) and **4** (d).

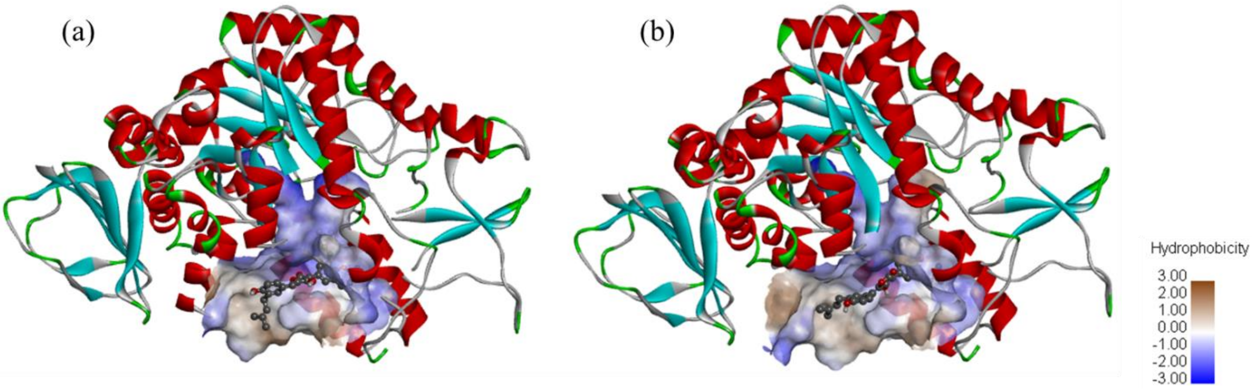

Figure S6. Full view of dock conformation of the isomaltase (3A4A) to inhibitors **1** (a) or **4** (b).

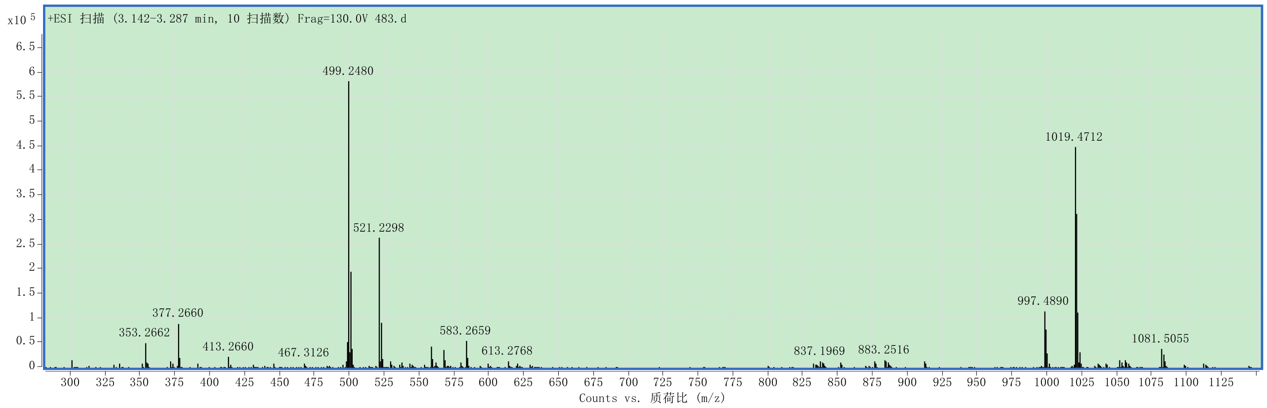

Figure S7. HRESIMS data of compound **1**

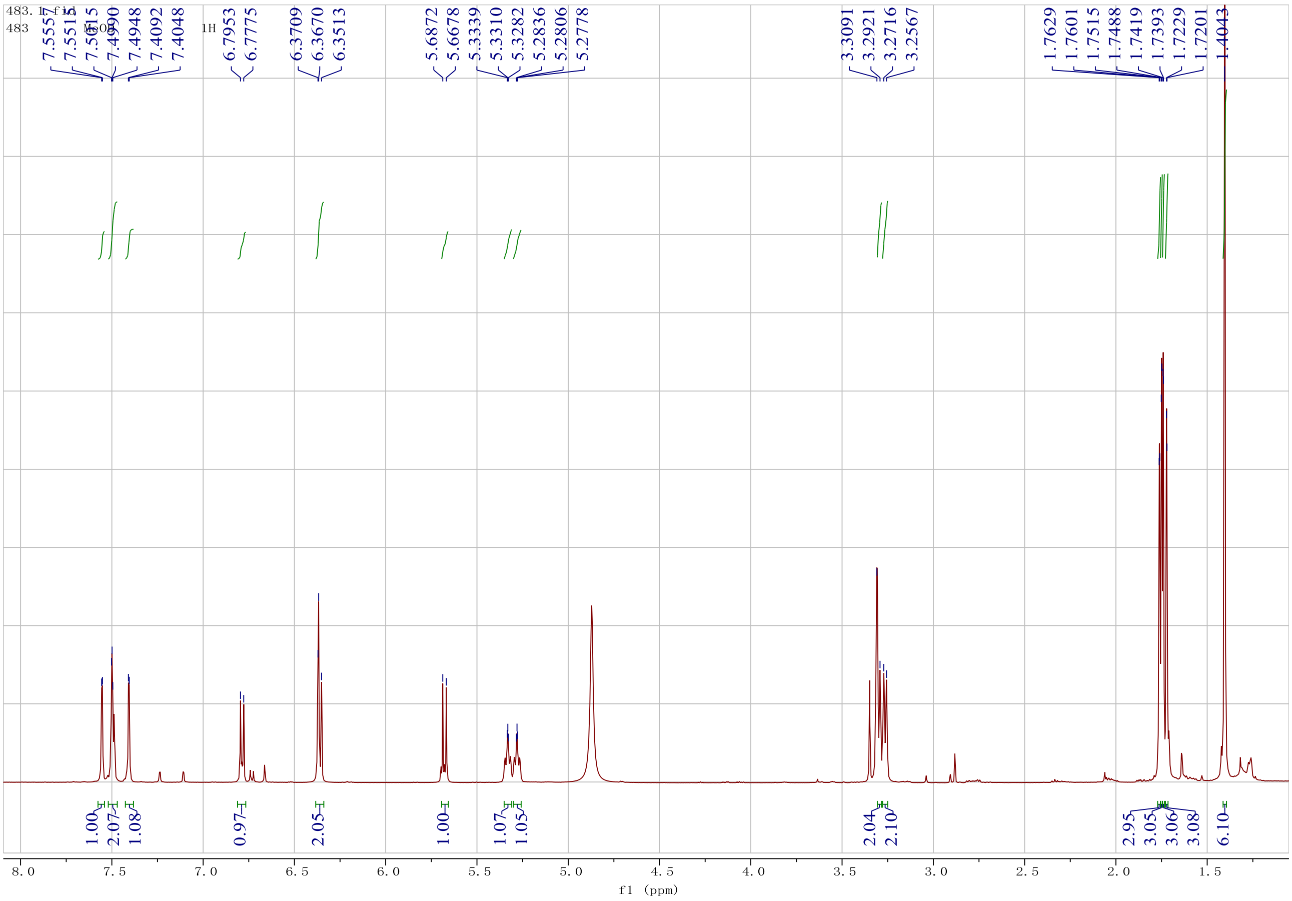

Figure S8. ^{1}H-NMR spectrum of compound **1** in CD_3OD

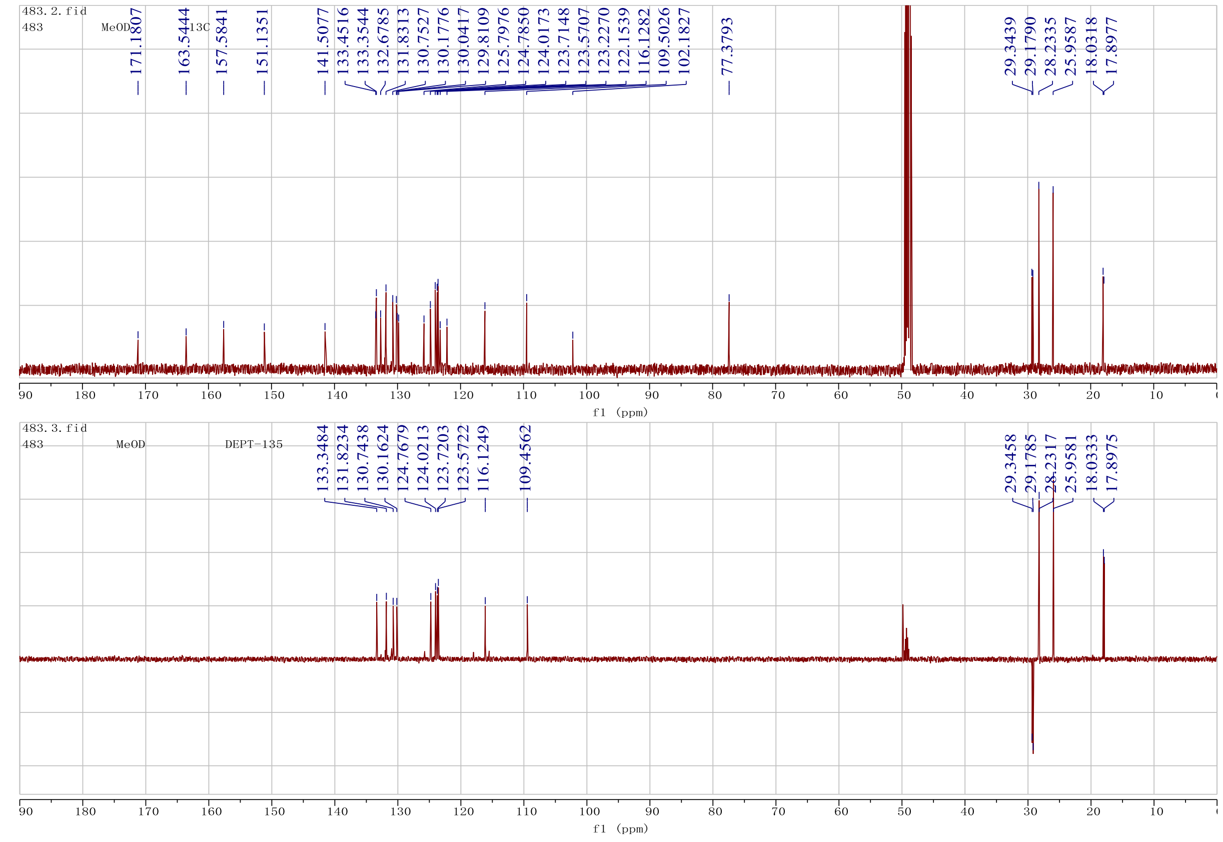

Figure S9. ^{13}C-NMR and DEPT-135 spectra of compound **1** in CD_3OD

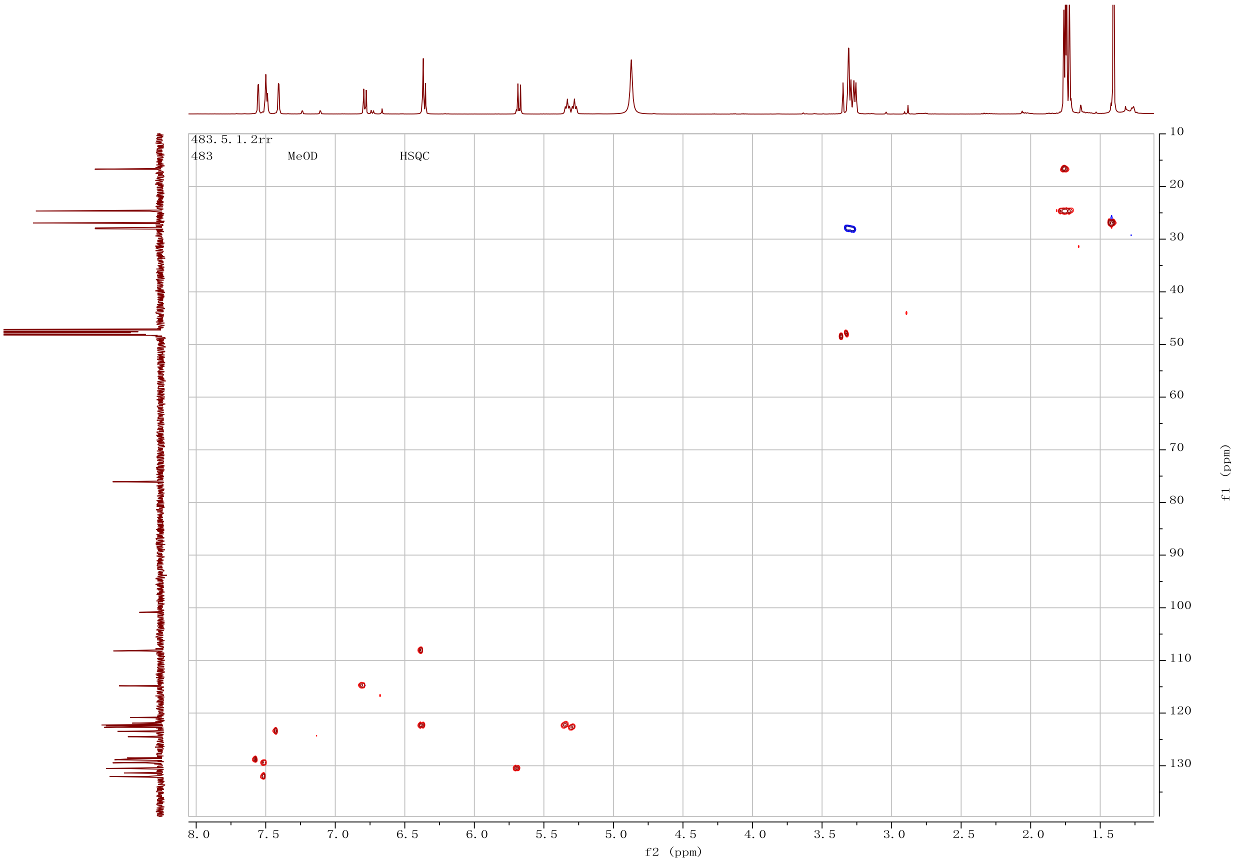

Figure S10. HSQC spectrum of compound **1** in CD_3OD

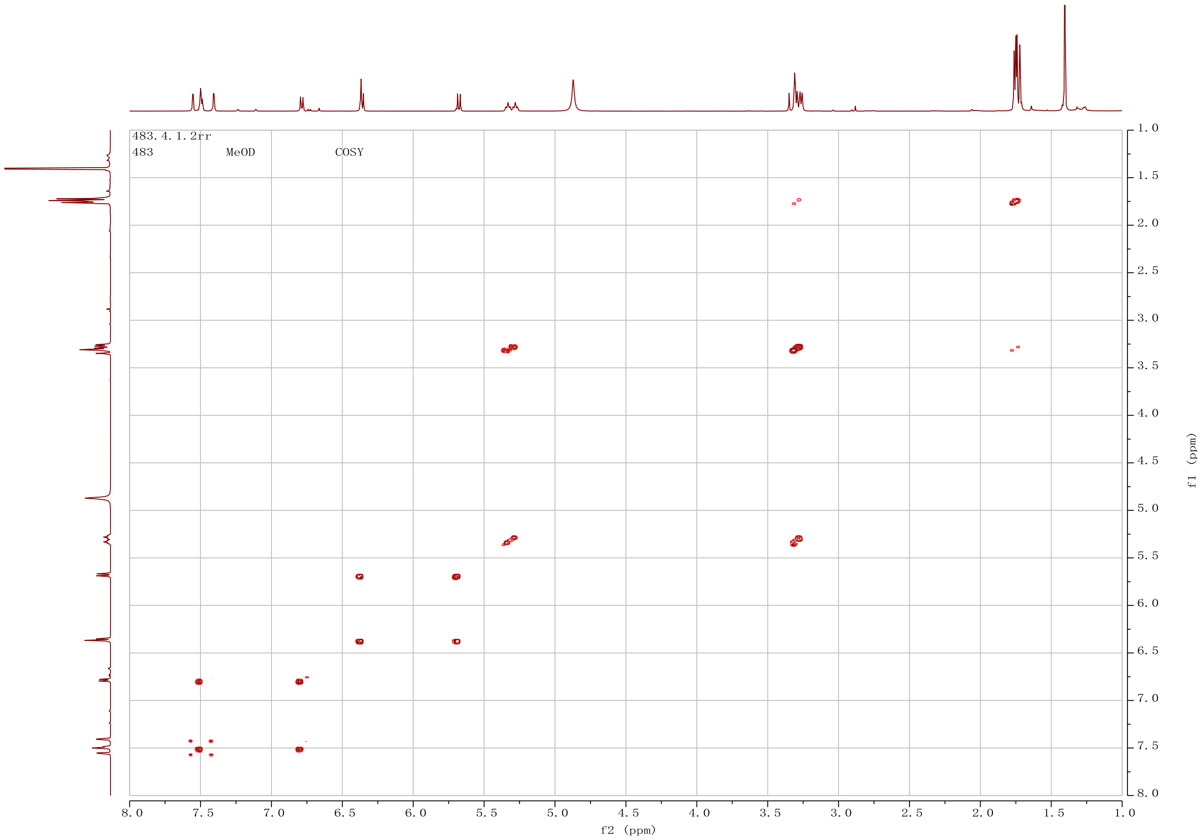

Figure S11. ^{1}H-^{1}H COSY spectrum of compound **1** in CD_3OD

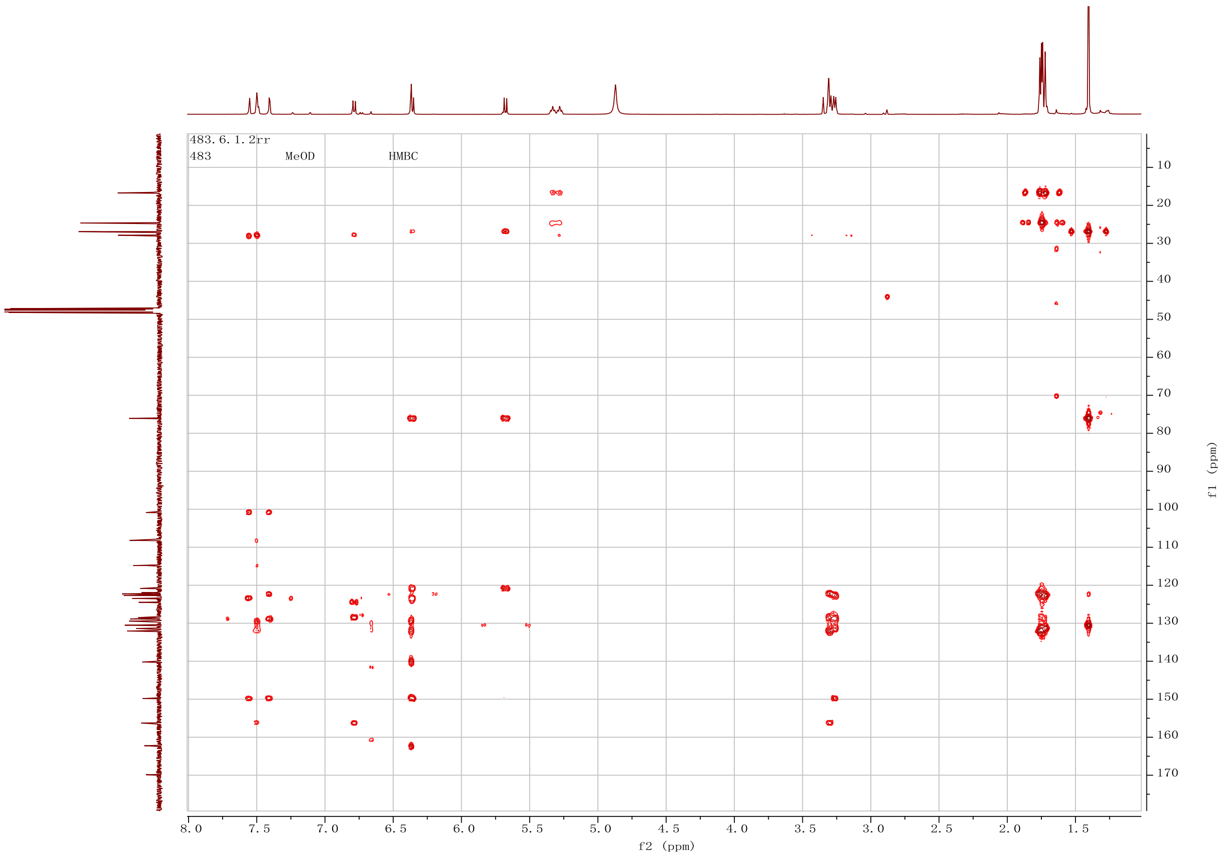

Figure S12. HMBC spectrum of compound **1** in CD_3OD

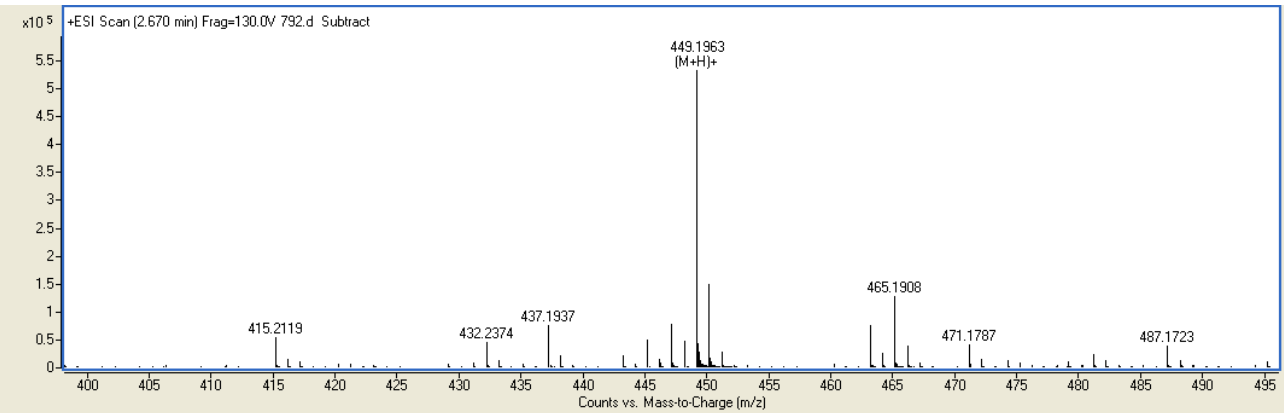

Figure S13. HRESIMS data of compound **2**

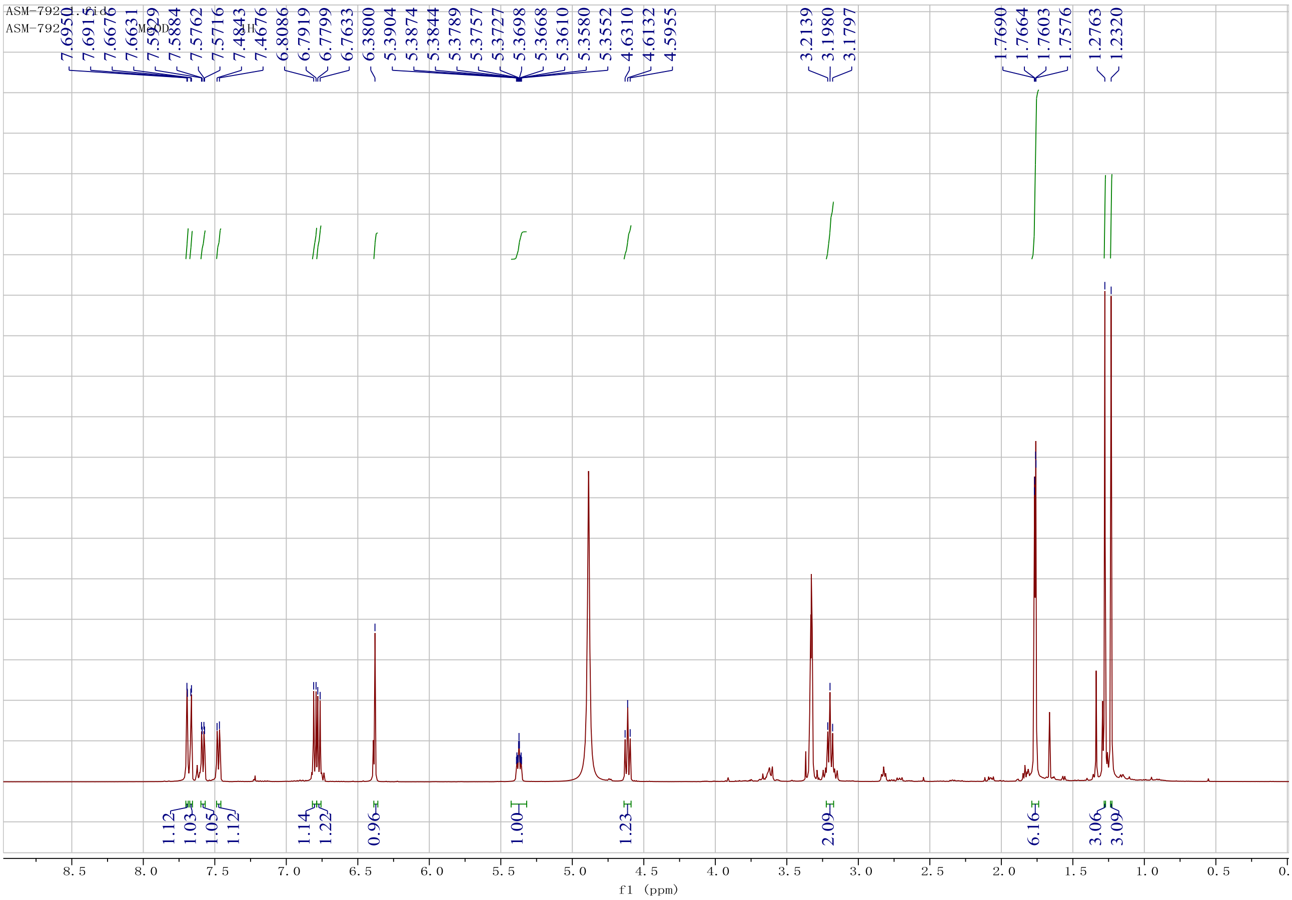

Figure S14. ^{1}H-NMR spectrum of compound **2** in CD_3OD

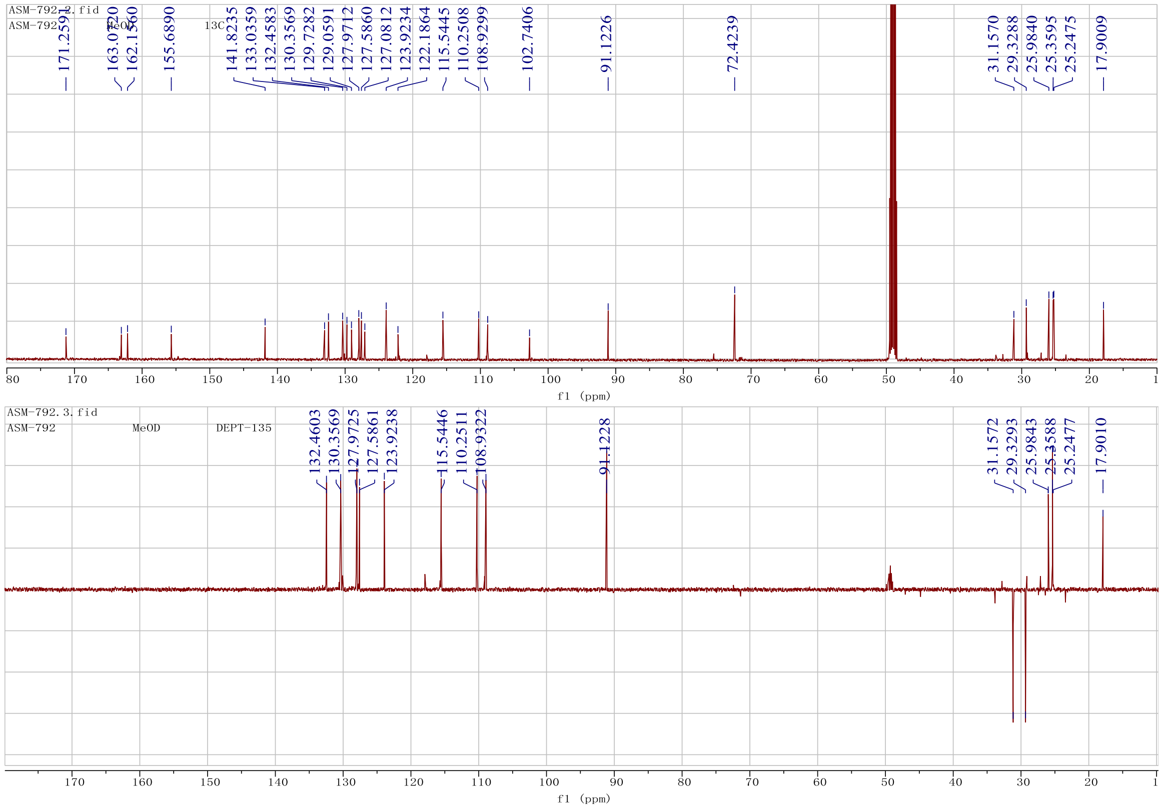

Figure S15. ^{13}C-NMR and DEPT-135 spectra of compound **2** in CD_3OD

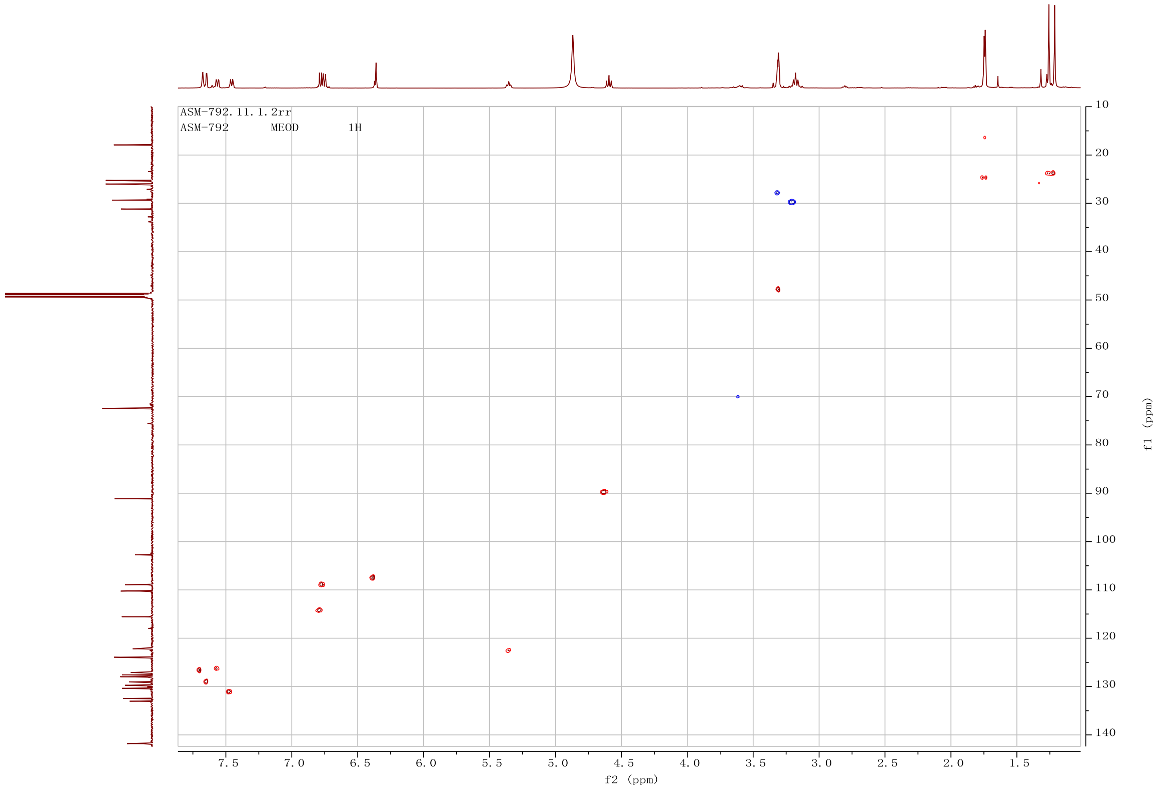

Figure S16. HSQC spectrum of compound **2** in CD_3OD

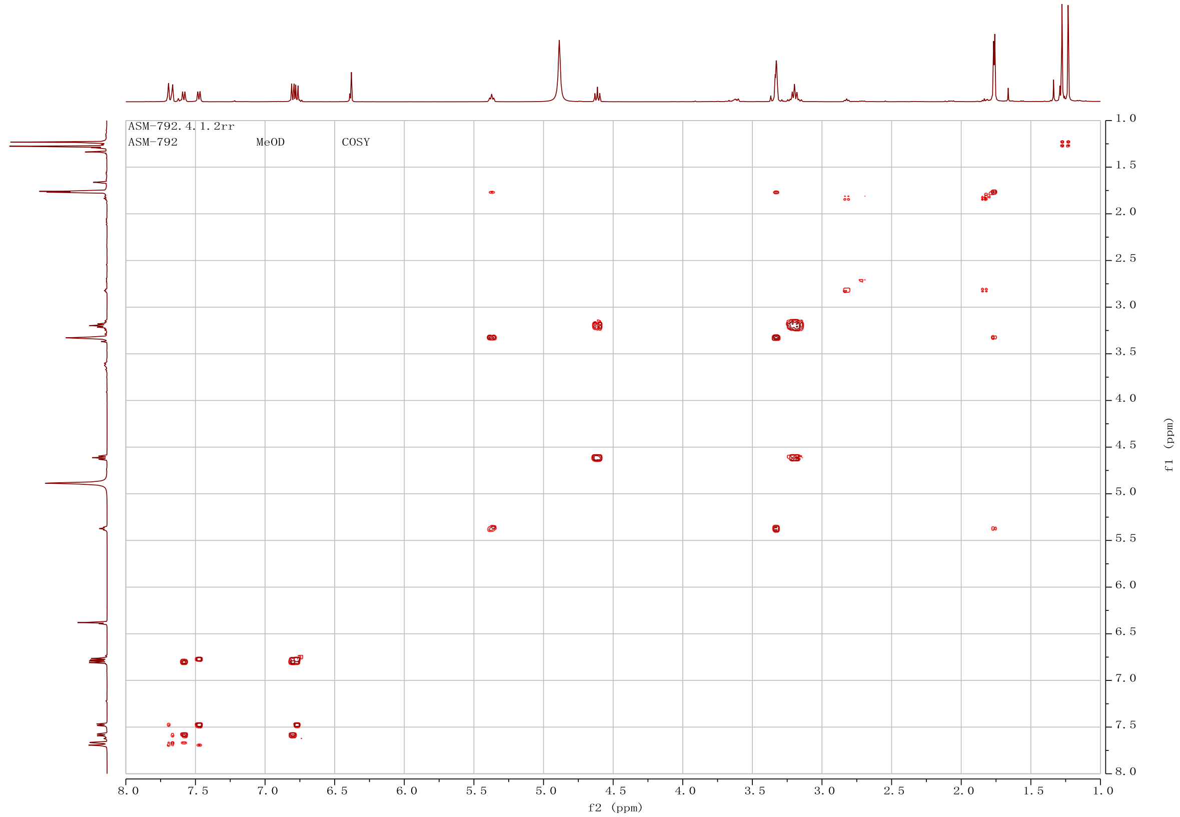

Figure S17. ^{1}H-^{1}H COSY spectrum of compound **2** in CD_3OD

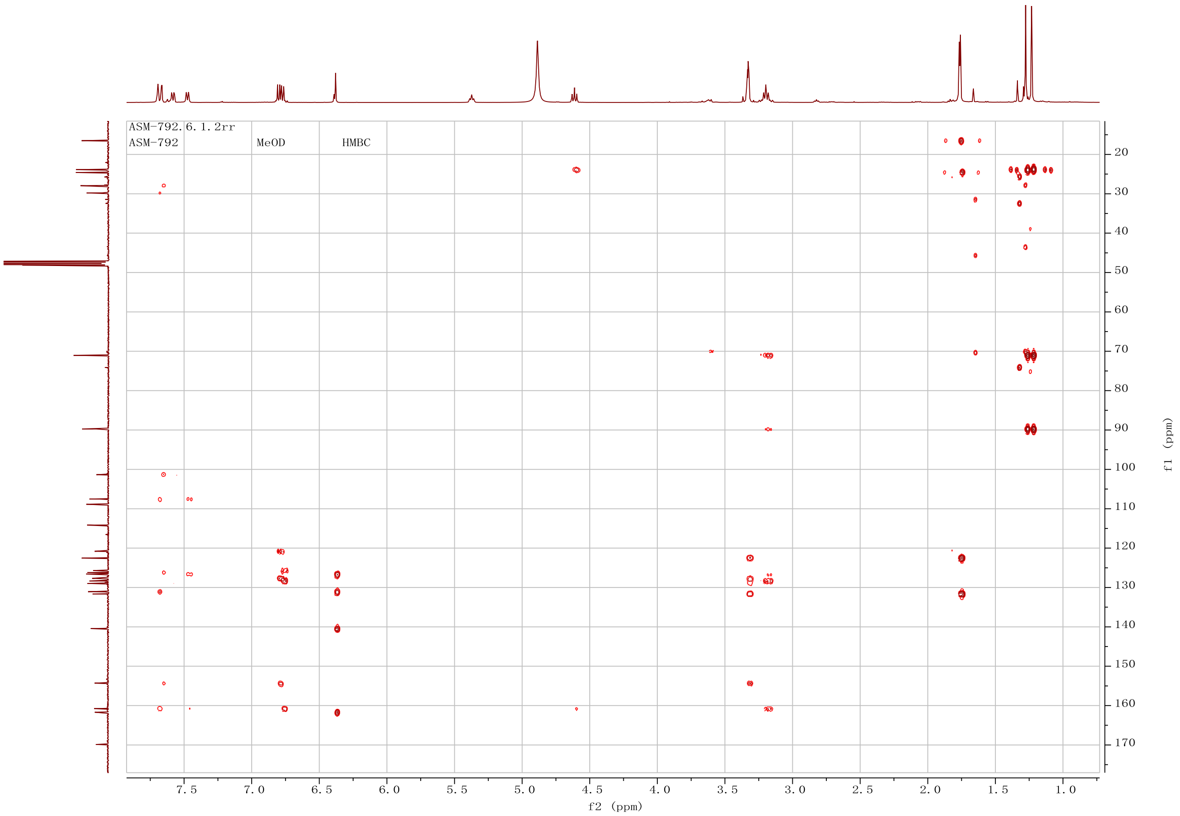

Figure S18. HMBC spectrum of compound **2** in CD_3OD

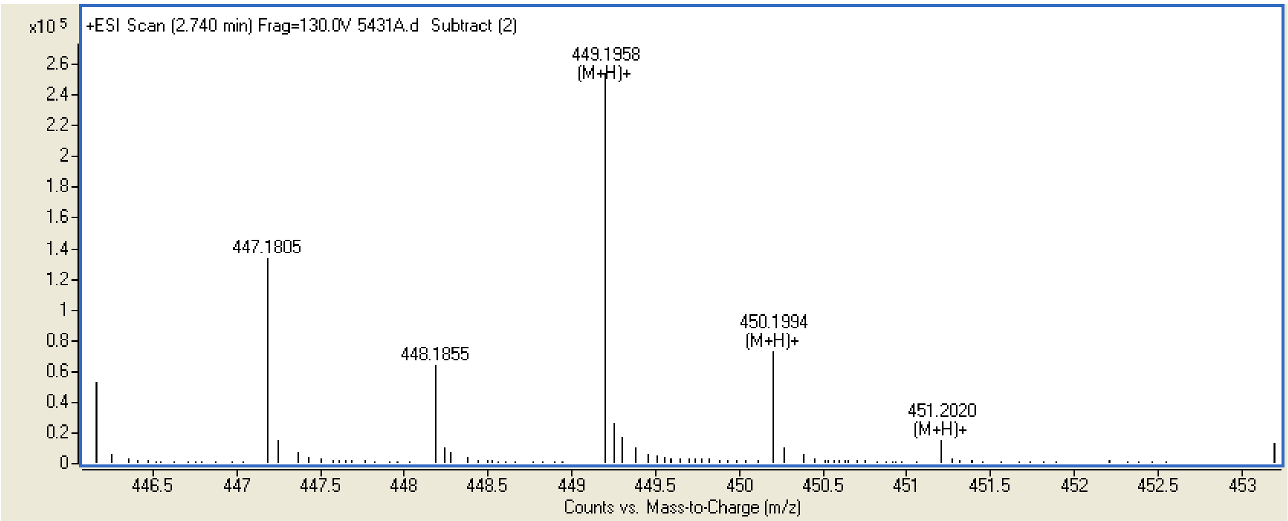

Figure S19. HRESIMS data of compound **3**

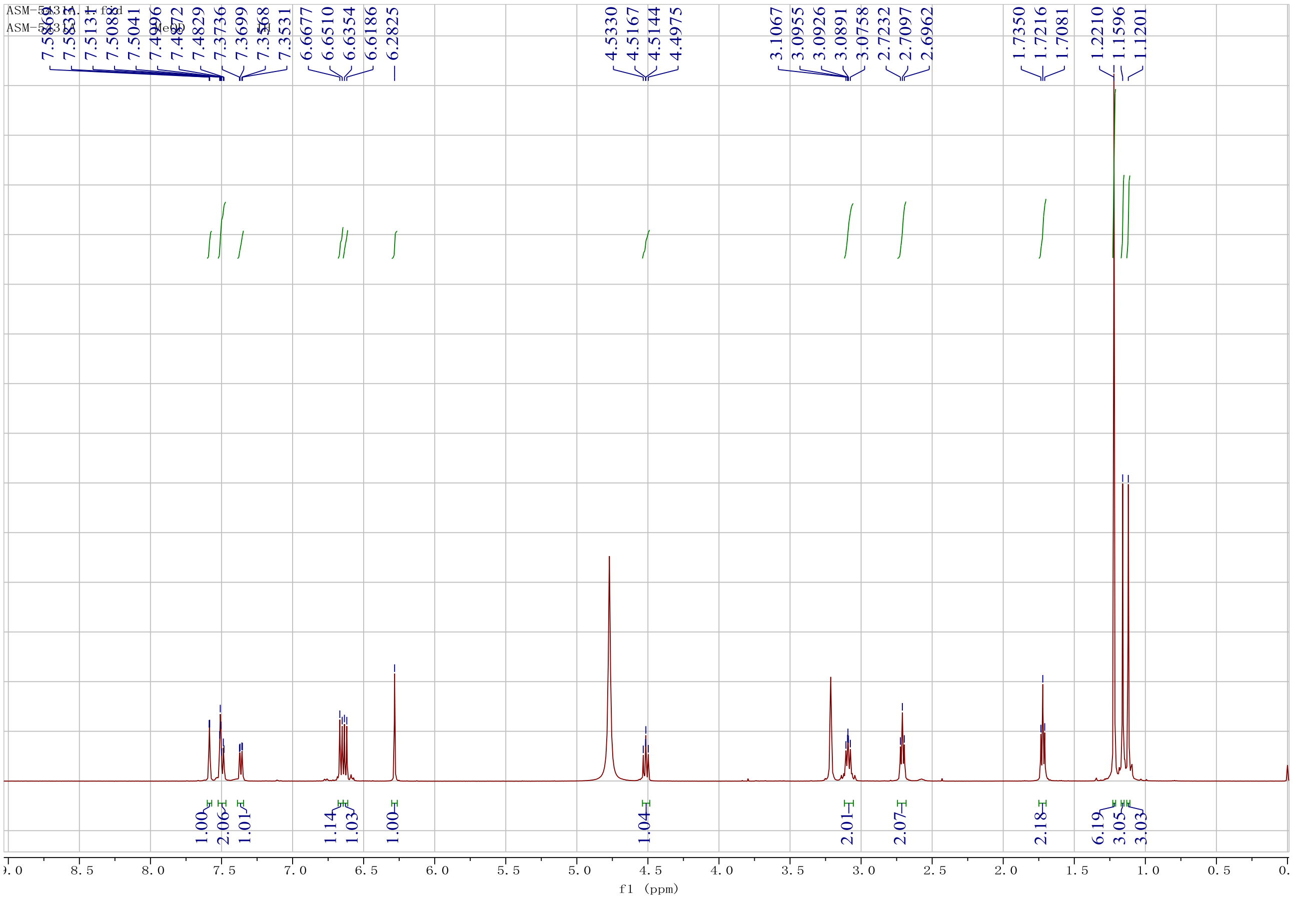

Figure S20. ^{1}H-NMR spectrum of compound **3** in CD_3OD

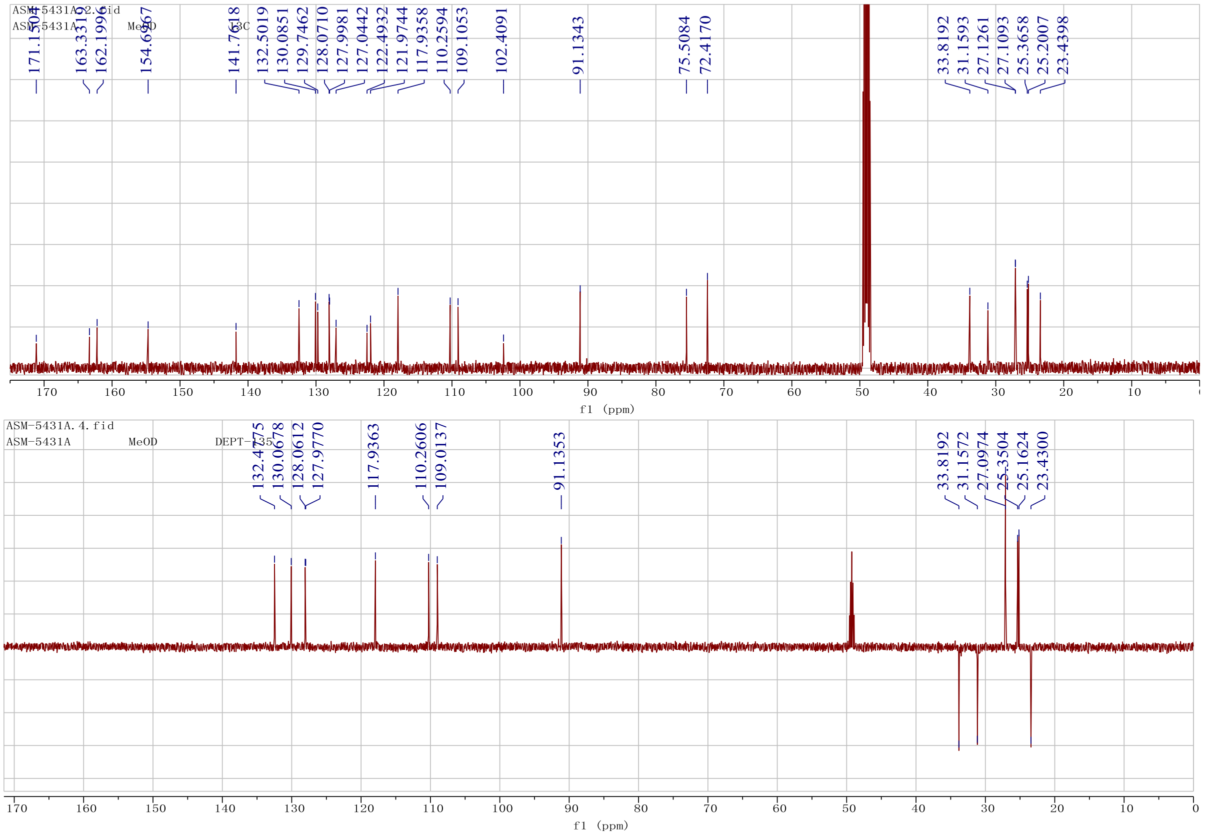

Figure S21. ^{13}C-NMR and DEPT-135 spectra of compound **3** in CD_3OD

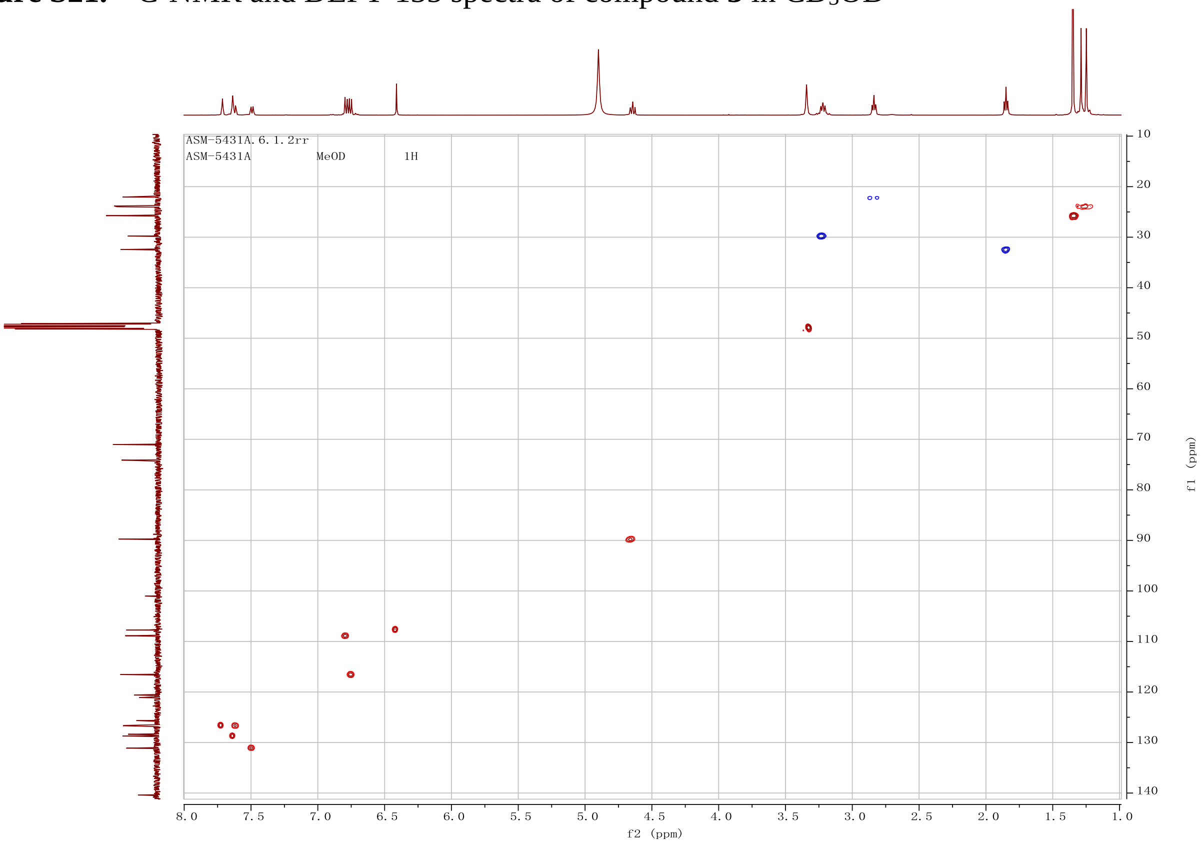

Figure S22. HSQC spectrum of compound **3** in CD_3OD

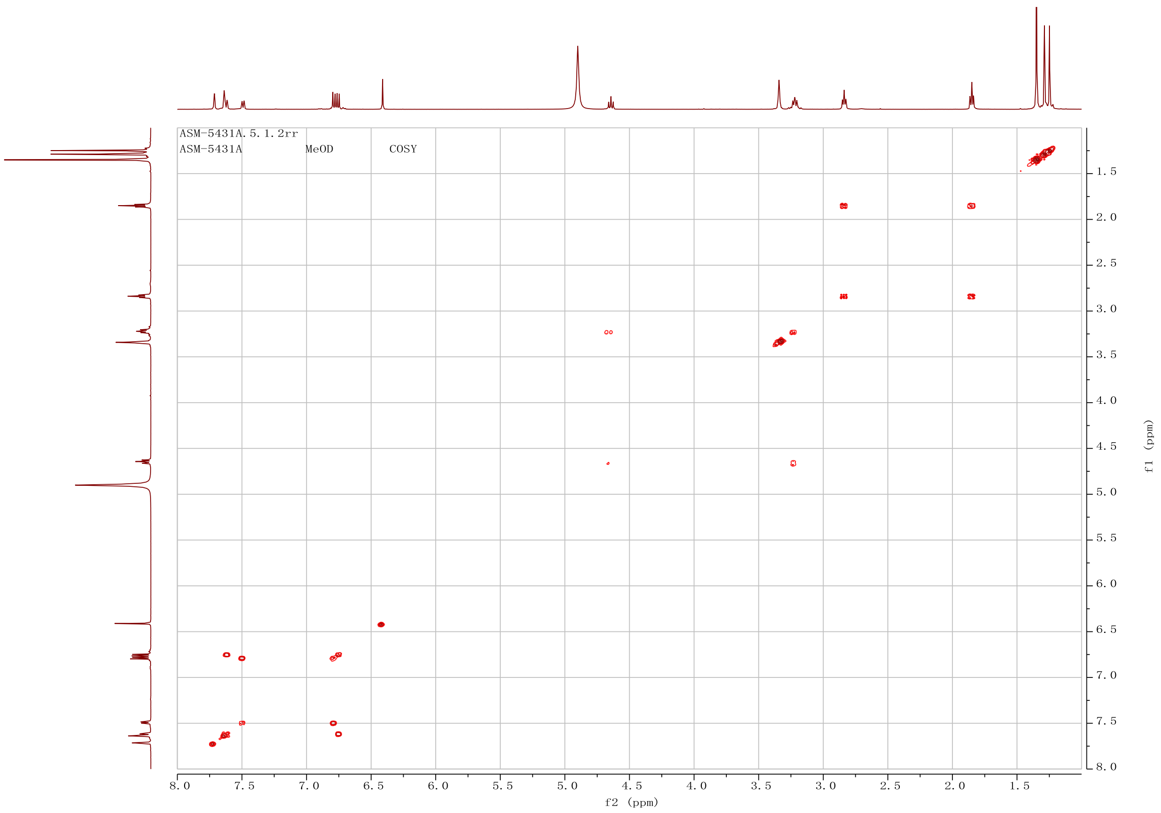

Figure S23. ^{1}H-^{1}H COSY spectrum of compound **3** in CD_3OD

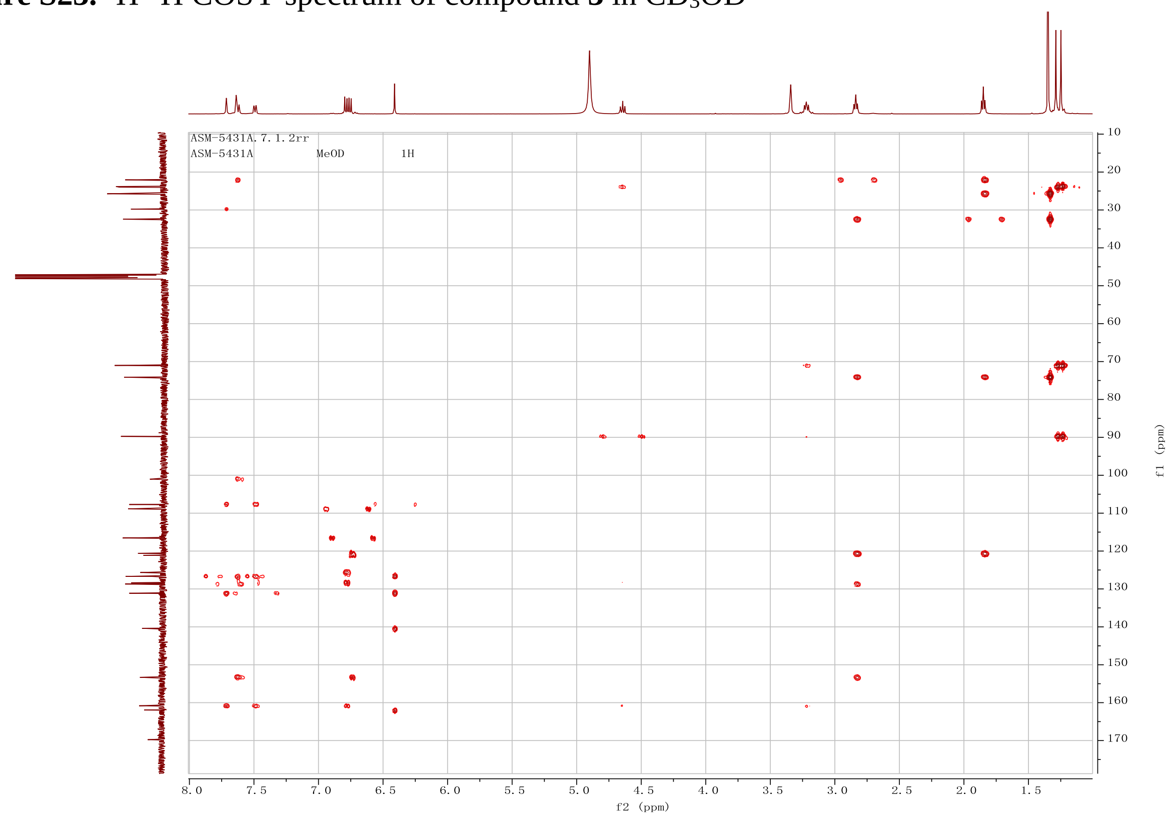

Figure S24. HMBC spectrum of compound **3** in CD_3OD

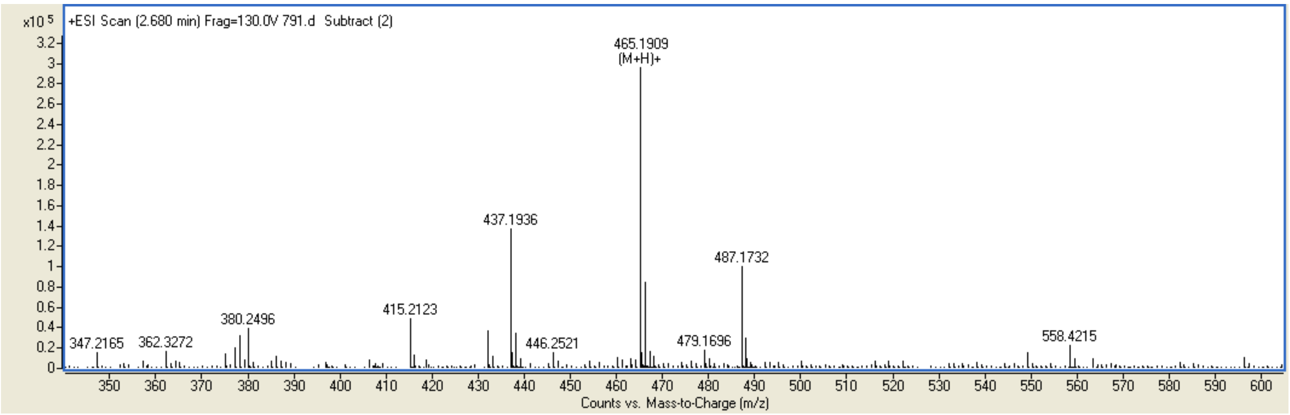

Figure S25. HRESIMS data of compound **5**

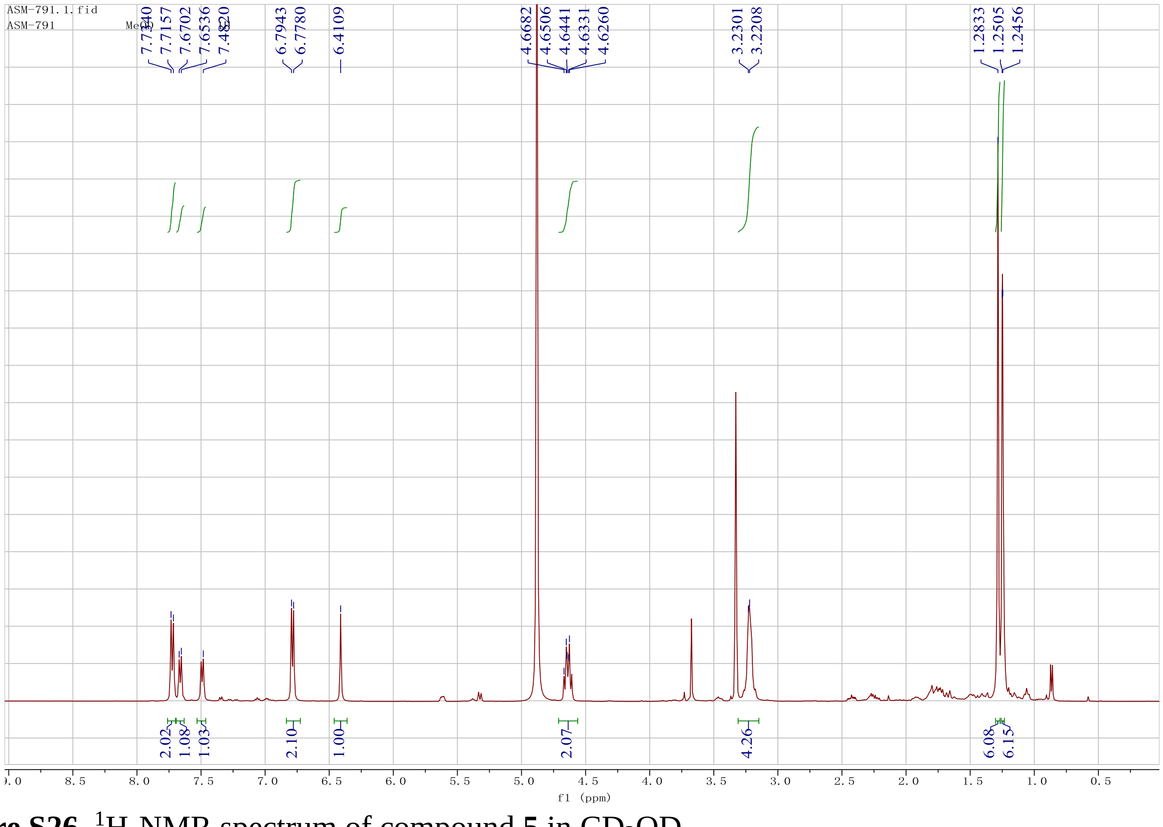

Figure S26. ^{1}H-NMR spectrum of compound **5** in CD_3OD

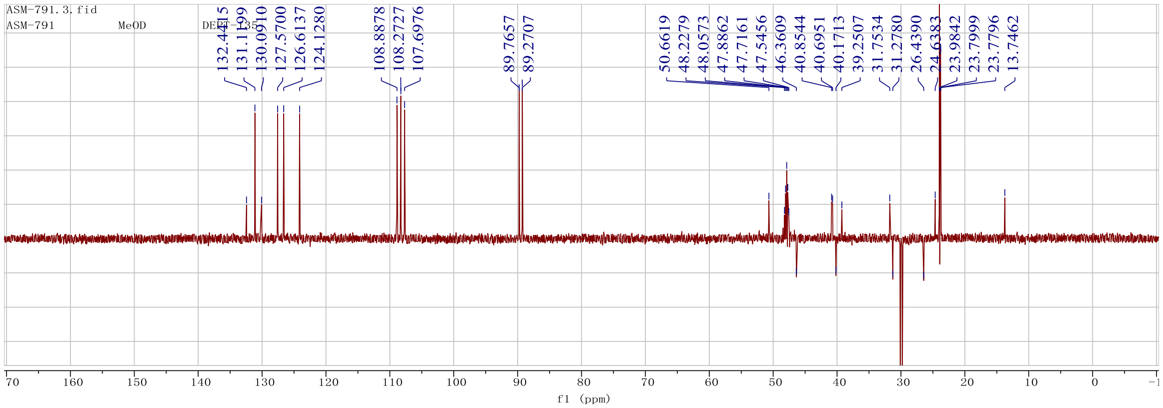

Figure S27. DEPT-135 spectrum of compound **5** in CD_3OD

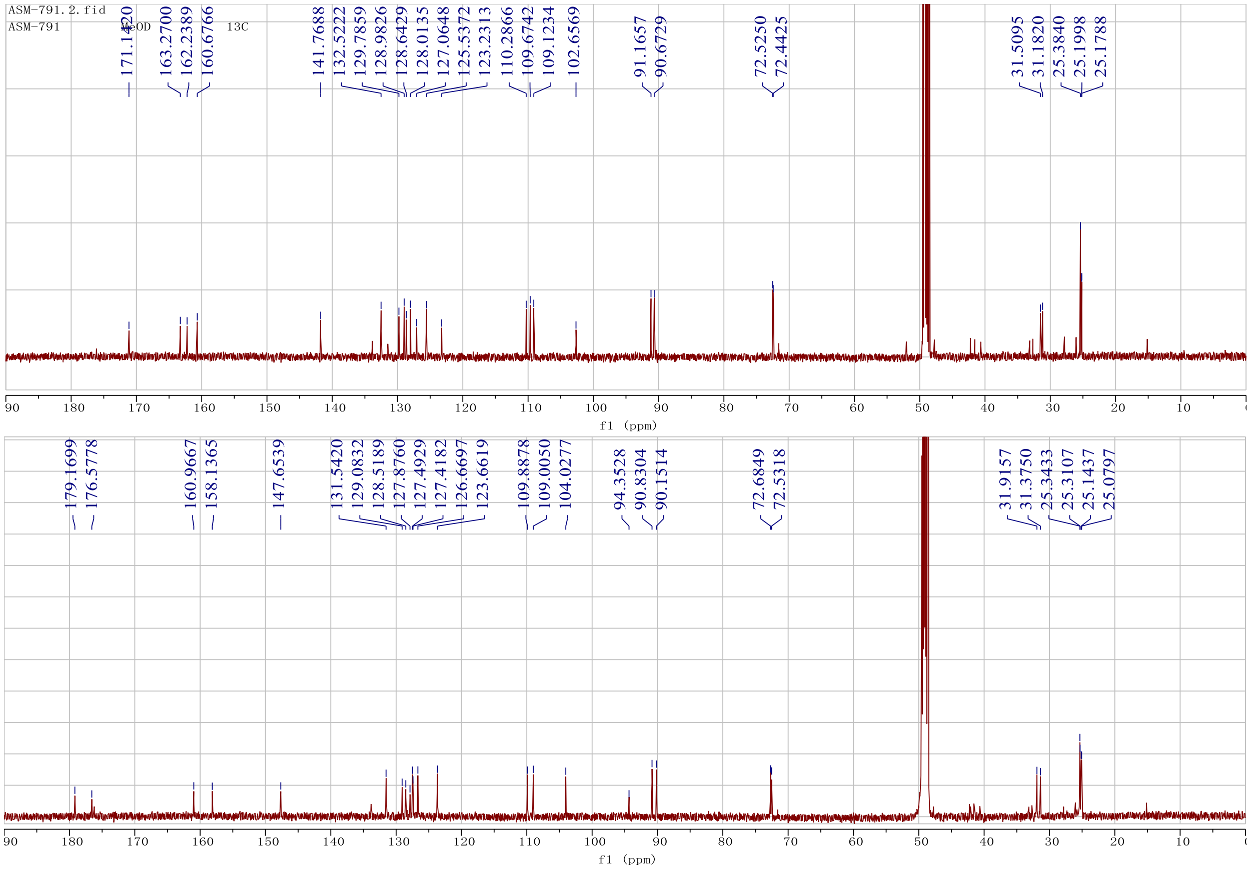

Figure S28. ^{13}C-NMR spectra of compound **5** (upper) and **5 sodium** (lower) in CD_3OD

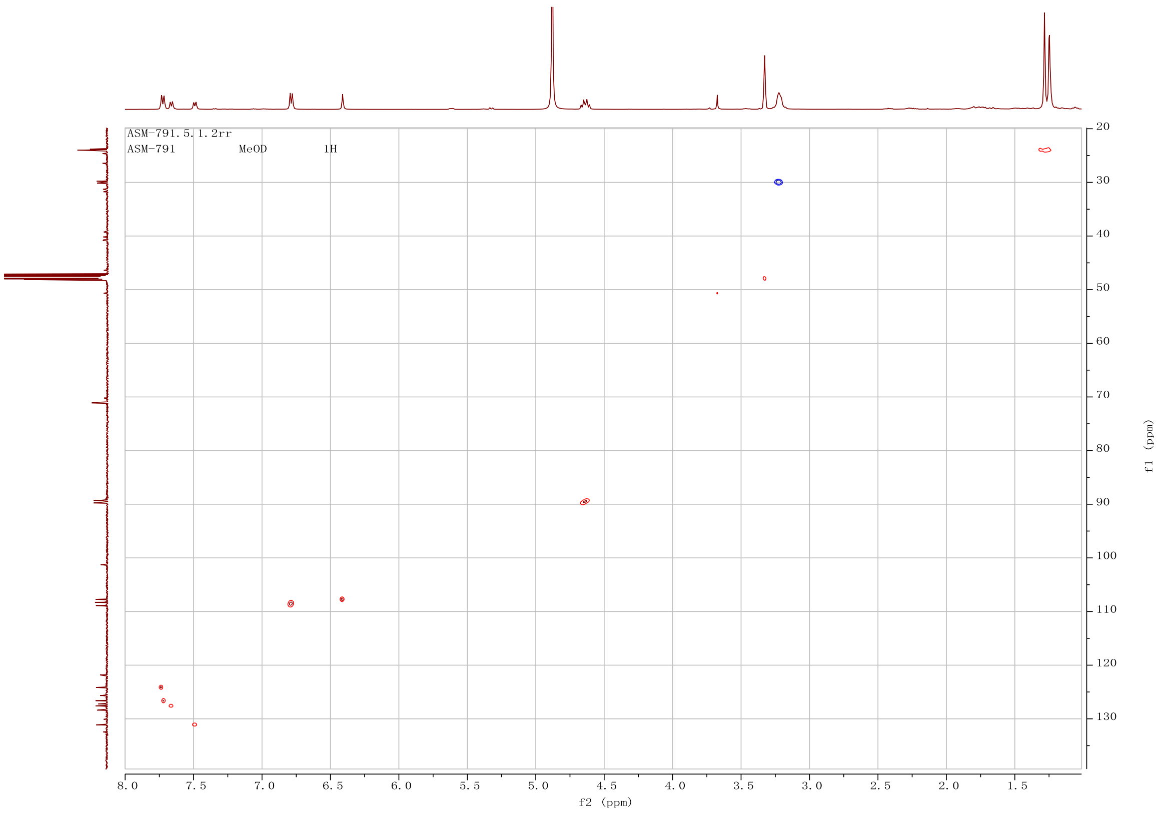

Figure S29. HSQC spectrum of compound **5** in CD_3OD

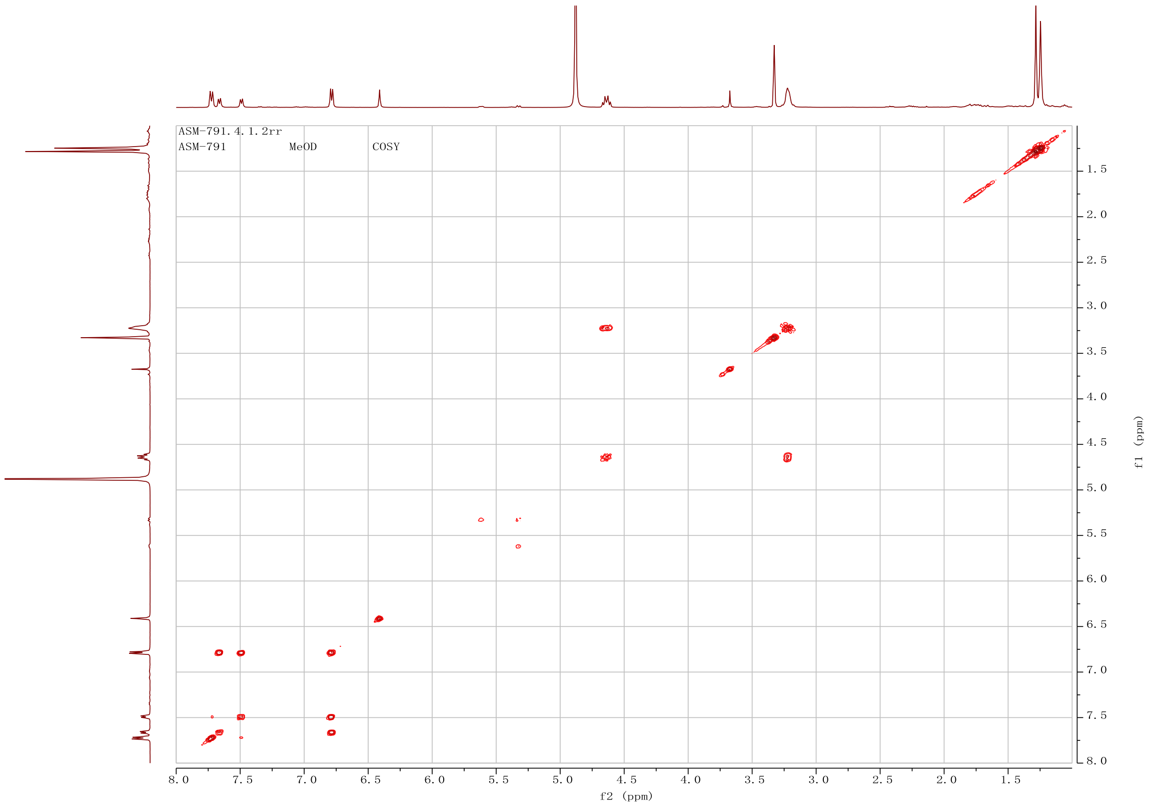

Figure S30. ^1H-^1H COSY spectrum of compound **5** in CD_3OD

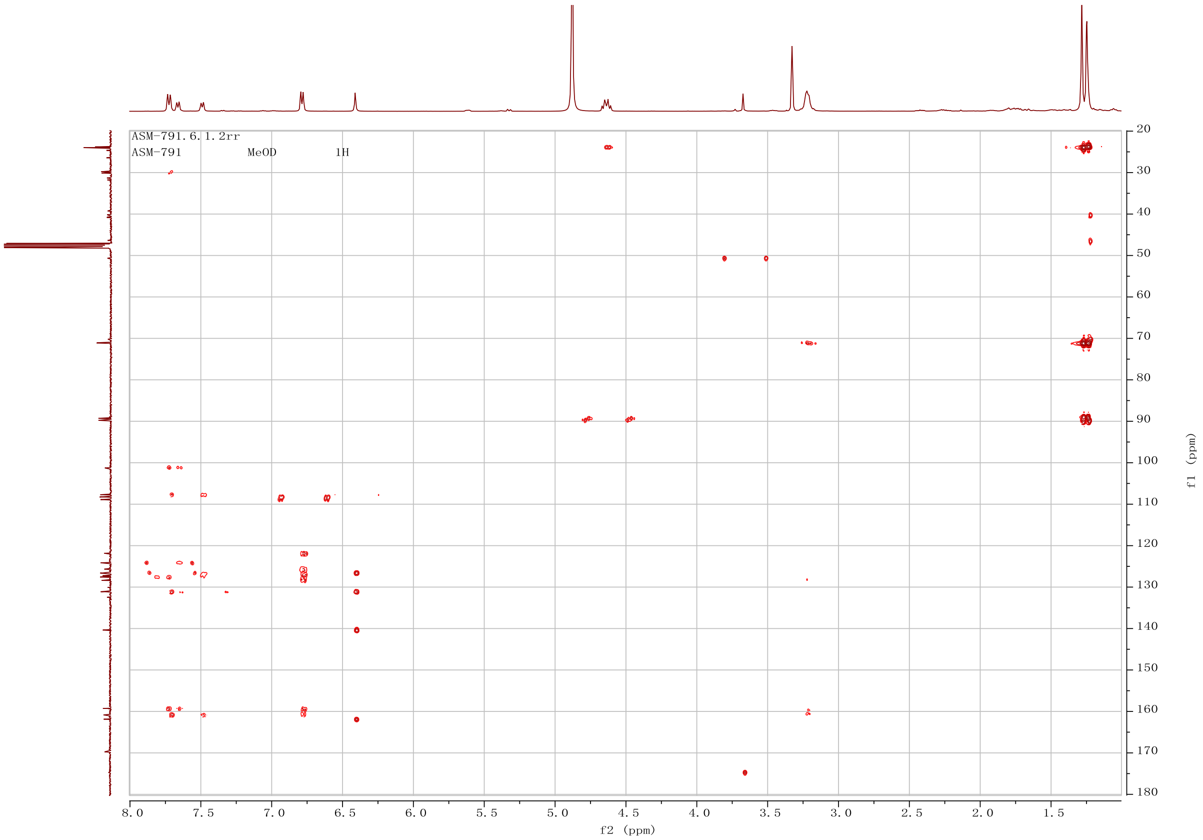

Figure S31. HMBC spectrum of compound **5** in CD_3OD
